# Noise and Dissipation on Coadjoint Orbits

**DOI:** 10.1007/s00332-017-9404-3

**Published:** 2017-07-17

**Authors:** Alexis Arnaudon, Alex L. De Castro, Darryl D. Holm

**Affiliations:** 1 Department of Mathematics, Imperial College, London, SW7 2AZ UK; 2 Departamento de Matemática, PUC-Rio, Rio de Janeiro, 22451-900 Brazil

**Keywords:** Stochastic geometric mechanics, Euler-Poincaré theory, Coadjoint orbits, Invariant measures, Random attractors, Lyapunov exponents, 37H10, 37J15, 60H10

## Abstract

We derive and study stochastic dissipative dynamics on coadjoint orbits by incorporating noise and dissipation into mechanical systems arising from the theory of reduction by symmetry, including a semidirect product extension. Random attractors are found for this general class of systems when the Lie algebra is semi-simple, provided the top Lyapunov exponent is positive. We study in details two canonical examples, the free rigid body and the heavy top, whose stochastic integrable reductions are found and numerical simulations of their random attractors are shown.

## Introduction

Geometric mechanics, introduced in Poincaré (1901), is a powerful formalism for understanding dynamical systems whose Lagrangian and Hamiltonian are invariant under the transformations of the configuration manifold *M* by a Lie group *G*. Examples of its applications range from the simple finite dimensional dynamics of the freely rotating rigid body to the infinite dimensional dynamics of the ideal fluid equations. For a historical review and basic references, see, e.g., Holm et al. (1998). See Marsden and Ratiu (1999), Holm (2008) and Holm et al. (2009) for textbook introductions to geometric mechanics and background references. One of the main approaches of geometric mechanics is the method of reduction of the motion equations of a mechanical system by a Lie group symmetry *G* in either its Lagrangian formulation on the tangent space *TM* of a configuration manifold *M*, or its Hamiltonian formulation on the cotangent space $T^*M$.

The method of reduction by symmetry yields reduced Lagrangian and Hamiltonian formulations of the Euler–Poincaré equations governing the dynamics of the momentum map $J: T^*M\rightarrow \mathfrak {g}^*$, where $\mathfrak {g}^*$ is the dual Lie algebra of the Lie symmetry group *G*. In general terms, Lie group reduction by symmetry simplifies the motion equations of a mechanical system with symmetry by transforming them into new dynamical variables in $\mathfrak {g}^*$ which are invariant under the same Lie group symmetries as the Lagrangian and Hamiltonian of the dynamics. More specifically, on the Lagrangian side, the new invariant variables under the Lie symmetries are obtained from Noether’s theorem, via the tangent lift of the infinitesimal action of the Lie symmetry group on the configuration manifold. The unreduced Euler–Lagrange equations are replaced by equivalent Euler–Poincaré equations expressed in the new invariant variables in $\mathfrak {g}^*$, plus an auxiliary reconstruction equation, which restores the information in the tangent space of the configuration space lost in transforming to group invariant dynamical variables. On the Hamiltonian side, after a Legendre transformation, equivalent new invariant variables in $\mathfrak {g}^*$ are defined by the momentum map $J:T^*M\rightarrow \mathfrak {g}^*$ from the phase-space $T^*M$ of the original system on the configuration manifold *M* to the dual $\mathfrak {g}^*$ of the Lie symmetry algebra $\mathfrak {g}\simeq T_eG$, via the cotangent lift of the infinitesimal action of the Lie symmetry group on the configuration manifold. The cotangent lift momentum map is an equivariant Poisson map which reformulates the canonical Hamiltonian flow equations in phase space as noncanonical Lie–Poisson equations governing flow of the momentum map on an orbit of the coadjoint action of the Lie symmetry group on the dual of its Lie algebra $\mathfrak {g}^*$, plus an auxiliary reconstruction equation for lifting the Lie group reduced coadjoint motion back to phase space $T^*M$.

Thus, Lie symmetry reduction yields coadjoint motion of the corresponding momentum map. The dimension of the dynamical system reduces because its solutions are restricted to remain on certain subspaces of the original phase space, called coadjoint orbits. These are orbits of the action of the group *G* on $\mathfrak {g}^*$, the dual space of its Lie algebra $\mathfrak {g}$. Coadjoint orbits lie on level sets of the distinguished smooth functions $C\in \mathcal {F}:\mathfrak {g}^*\rightarrow \mathbb {R}$ of the symmetry-reduced dual Lie algebra variables $\mu \in \mathfrak {g}^*$ called Casimir functions. Thus, the Casimir functions are conserved quantities. Indeed, Casimir functions have null Lie–Poisson brackets $\{C,F\}(\mu )=0$ with any other functions $F\in \mathcal {F}(\mathfrak {g}^*)$, including the reduced Hamiltonian $h(\mu )$. Furthermore, level sets of the Casimirs, on which the coadjoint orbits lie, are symplectic manifolds which provide the framework on which geometric mechanics is constructed. These symplectic manifolds have many applications in physics, as well as in symplectic geometry, whenever Lie symmetries are present. In particular, coadjoint motion of the momentum map $J(t)=\mathrm{Ad}^*_{g(t)}J(0)$ for a solution curve $g(t)\in C(G)$ takes place on the intersections of level sets of the Casimirs with level sets of the Hamiltonian.

Given this framework for Lie group reduction by symmetry in deterministic geometric mechanics, we seek strategies for adding stochasticity and dissipation in classical mechanical systems with symmetry which preserve the coadjoint motion structure of the unperturbed deterministic dynamics. Specifically, we seek stochastic coadjoint motion equations whose solutions do not preserve the energy while lying on the coadjoint orbits of the unperturbed equation. Consequently, our first goal in this paper will be to replace the deterministic equations for coadjoint motion by stochastic processes whose solutions lie on coadjoint orbits. However, simply inserting additive noise into the deterministic equations will not, in general, produce coadjoint motion on level sets of the Casimirs of a Lie–Poisson bracket. Instead, our approach in developing a systematic derivation of stochastic deformations that preserve coadjoint orbits will be to constrain the variations in Hamilton’s principle to preserve the transport relations for infinitesimal transformations defined by the action of a stochastic vector field on the configuration manifold, as in Holm (2015).

Having used the constrained Hamilton’s principle to derive the stochastic coadjoint motion equation, the study of the associated Fokker–Planck equation and its invariant measure will follow naturally, and be well defined, at least provided one restricts to finite dimensional mechanical systems. The resulting Fokker–Planck equation defines a probability density for coadjoint motion on Casimir surfaces since it takes the form of a Lie–Poisson equation for the transport part, and a double Lie–Poisson structure for the diffusion part, both of which generate motion along coadjoint orbits. As we will discover, this form of the Fokker–Planck equation in the absence of any additional energy dissipation will imply that the invariant measure (asymptotically in time) simply tends to a constant on Casimir surfaces.

Next, we shall include an energy dissipation mechanism, called double bracket dissipation, or selective decay, which preserves the coadjoint orbits, while it decays the energy towards its minimum value, usually associated with an equilibrium state of the deterministic system. We refer to Bloch et al. (1996), Gay-Balmaz and Holm (2013) and Gay-Balmaz and Holm (2014) and references therein for complete studies of double bracket dissipations. In a second step, we will include this double bracket dissipative term in our stochastic coadjoint motion equations and again study the associated Fokker–Planck equation and its invariant measure, which will no longer be constant. Instead, the invariant measure will be an exponential function of the energy (Gibbs measure).

The procedure we shall follow will produce stochastic dissipative dynamical systems on coadjoint orbits. The study of multiplicative noise and nonlinear dissipation in these systems is greatly facilitated by the geometric structure of the equations for coadjoint motion. Indeed, a large part of standard dynamical system theory will still apply in our setting. In particular, the proof of the existence of random attractors will follow a standard approach. We will mainly focus on this particular feature of random attractors of our systems, as it is an important diagnostic and has recently been an active field of research. The main idea behind the random attractor is the decomposition of the stationary distribution of the Fokker–Planck equation into random measures, called Sinai-Ruelle-Bowen, or SRB measures, whose expectation recovers the stationary distribution of the Fokker–Planck equation. See, e.g., Young (2002) for a short insightful review. Random attractors can also help in understanding the notion of reliability in complex dynamical systems, see for example (Lin et al. 2009). These ideas have recently been developed and applied actively in the context of climate studies. For example, see Chekroun et al. (2011) for discussions and illustrations of how the notion of random SRB measures and random attractors enable the investigation of detailed geometric structures of the random attractors associated with nonlinear, stochastically perturbed systems. In particular, high-resolution numerical studies of two idealised models of interest for climate dynamics (the Jin97 ENSO model and the Lorenz63 model) are reported in Chekroun et al. (2011). The present work follows a similar line of investigation for a class of nonlinear, stochastically perturbed systems which exhibit coadjoint motion. The proof of existence of non-singular SRB measures requires some work, but it can be accomplished for our general class of mechanical systems written on semi-simple Lie algebras. Although geometric mechanics can also describe infinite dimensional systems such as fluid mechanics (Holm et al. 1998), we will only focus here on finite dimensional systems, and in particular on systems described by semi-simple Lie algebras. The natural non-degenerate and bi-invariant pairing admitted by semi-simple Lie algebras will facilitate the computations involved in proving our results, although some of the results may still apply more generally.

In the Euler–Poincaré theory, introducing a parameter into the Lagrangian or Hamiltonian which breaks the symmetry results in a semidirect product of groups acting on coset spaces representing the order parameters, or advected quantities, which are not invariant under the original symmetry group (Holm et al. 1998). We will apply the theory of stochastic deformations that preserve coadjoint orbits for a particular class of semidirect product systems whose advected quantities live in the underlying vector space of the Lie algebra ${\mathfrak {g}}$. With this particular structure, which can be viewed as a generalisation of the heavy top, we will be able to prove the existence of SRB measures, provided the top Lyapunov exponent is positive. Although much of the present theory may also apply to more general systems than we treat here, as a first investigation, we will show that these particular mechanical systems in geometric mechanics exhibit interesting random attractors on coadjoint orbits when both noise and a certain type of double bracket dissipation are included.

As illustrations, we will discuss in detail two canonical elementary examples in the science of stochastic dissipative geometric mechanics. These two examples are the rigid body and the heavy top, which are also the well-known canonical examples for understanding symmetry reduction for deterministic geometric mechanics (Marsden and Ratiu 1999; Holm 2008; Holm et al. 2009. As mentioned earlier, the extensions here to include stochasticity and dissipation which preserve coadjoint orbits may be regarded as natural counterparts for geometric mechanics of the standard nonlinear dissipative systems, such as the stochastic Lorenz systems, treated, e.g., in Chekroun et al. (2011), Kondrashov et al. (2015).

*Main contributions of this work* Section 2 uses the Clebsch approach of Holm (2015) to introduce noise into the Euler–Poincaré equation for the momentum map, including its extension for semidirect product Lie symmetry groups. By construction, the momentum map for the stochastic vector field is the same as that for the deterministic vector field, so the stochastic and deterministic Euler–Poincaré equations for the momentum map may be compared directly. Section 3 introduces the selective decay mechanism for dissipation and studies the existence of random attractors. The first example of the Euler–Poincaré equation is treated in Sect. 5 with the free rigid body. Section 6 treats the Heavy Top as an example of the semidirect product extension. Finally, Sect. 7 briefly sketches the treatments of two other examples, the *SO*(4) free rigid body and the spring pendulum.

## Structure Preserving Stochastic Mechanics

Stochastic Hamilton equations were introduced along parallel lines with the deterministic canonical theory in Bismut (1982). These results were later extended to include reduction by symmetry in Lázaro-Camí and Ortega (2008). Reduction by symmetry of expected-value stochastic variational principles for Euler–Poincaré equations was developed in Arnaudon et al. (2014) and Chen et al. (2015). Stochastic variational principles were also used in constructing stochastic variational integrators in Bou-Rabee and Owhadi (2009).

The inclusion of noise in fluid equations has a long history in the scientific literature. For reviews and recent advances in stochastic turbulence models, see Kraichnan (1994) and Gawedzki and Kupiainen (1996); and in the analysis of stochastic Navier-Stokes equations, see Mikulevicius and Rozovskii (2001). These studies of the stochastic Navier-Stokes equation are fundamental in the analysis of fluid turbulence. Expected-value stochastic variational principles leading to the derivation of the Navier-Stokes motion equation for incompressible viscous fluids have been investigated in Arnaudon and Cruzeiro (2012). For further references, we refer to Holm (2015), on which the present work is based. This work has recently had a sequence of further developments, which we now briefly sketch.

1. In Holm (2015), the extension of geometric mechanics to include stochasticity in nonlinear fluid theories was accomplished by using Hamilton’s variational principle, constrained to enforce stochastic Lagrangian fluid trajectories arising from the stochastic Eulerian vector field
  2.1 $$\begin{aligned} v(x,t,dW) := u(x,t)\,\hbox {d}t + \sum _{i=1}^N \xi _i (x) \circ dW^i(t) \,, \end{aligned}$$
  regarded as a decomposition into the sum of a drift velocity *u*(*x*, *t*) and a sum over stochastic terms. Imposing this decomposition as a constraint on the variations in Hamilton’s principle for fluid dynamics (Holm et al. 1998), led in Holm (2015) to applications in a variety of fluid theories, particularly for geophysical fluid dynamics (GFD).
2. The same stochastic fluid dynamics equations derived in Holm (2015) were also discovered in Cotter et al. (2017) to arise in a multi-scale decomposition of the deterministic Lagrange-to-Euler flow map, into a slow large-scale mean and a rapidly fluctuating small-scale map. Homogenisation theory was used to derive effective slow stochastic particle dynamics for the resolved mean part, thereby justifying the stochastic fluid partial equations in the Eulerian formulation. The application of rigorous homogenisation theory required assumptions of mildly chaotic fast small-scale dynamics, as well as a centring condition, according to which the mean of the fluctuating deviations was small, when pulled back to the mean flow.
  Paper (Cotter et al. 2017) justified regarding the Eulerian vector field in (2.1) as a genuine decomposition of the fluid velocity into a sum of drift and stochastic parts, rather than simply a perturbation of the dynamics meant to model unknown effects in uncertainty quantification. As a genuine decomposition of the solution, one should expect that the properties of the fluid equations with stochastic transport noise should closely track the properties of the unapproximated solutions of the fluid equations. For example, if the unapproximated model equations are Hamiltonian, then the model equations with stochastic transport noise should also be Hamiltonian, as shown in Holm (2015).
3. Paper (Crisan et al. 2017) showed that the same stochastic fluid dynamics derived in Holm (2015) naturally arises from an application of a stochastic Lagrange-to-Euler map to Newton’s second law for a Lagrangian domain of fluid, acted on by an external force. In addition, local well-posedness in regular spaces and a Beale-Kato-Majda blow-up criterion are proved in Crisan et al. (2017) for the stochastic model of the 3D Euler fluid equation for incompressible flow derived in Holm (2015). Thus, the analytical properties of the 3D Euler fluid equations with stochastic transport noise derived in Holm (2015) closely mimic the corresponding analytical properties of the original deterministic 3D Euler fluid equations.
4. Inspired by spatiotemporal observations from satellites of the trajectories of objects drifting near the surface of the ocean in the National Oceanic and Atmospheric Administration’s “Global Drifter Program”, paper (Gay-Balmaz and Holm 2017) developed data-driven stochastic models of geophysical fluid dynamics (GFD) with non-stationary spatial correlations representing the dynamical behaviour of oceanic currents. These models were derived using reduction by symmetry of stochastic variational principles, leading to stochastic Hamiltonian systems, whose momentum maps, conservation laws and Lie–Poisson bracket structures were used in developing the new stochastic Hamiltonian models of GFD with nonlinearly evolving stochastic properties.

The present section incorporates stochasticity into finite dimensional mechanical systems admitting Lie group symmetry reduction, by using the standard Clebsch variational method for deriving cotangent lifted momentum maps. We review the standard approach to Lie group reduction by symmetry for finite dimensional systems in 2.1 and incorporate noise into this approach in Sect. 2.2. Next, we describe the semidirect extension in 2.3 and study the associated Fokker–Planck equations and stationary distribution in 2.4. The primary examples from classical mechanics with symmetry will be the free rigid body and the heavy top under gravity.

### Euler–Poincaré Reduction

Classical mechanical systems with symmetry can often be understood geometrically in the context of Lagrangian or Hamiltonian reduction, by lifting the motion *m*(*t*) on the configuration manifold *M* to a Lie symmetry group via the action of the symmetry group *G* on the configuration manifold, by setting $m(t)=g(t)m(0)$, where the multiplication is to be understood as the action of *G* to *M*. This procedure lifts the solution of the motion equation from a curve $m(t)\in M$ to a curve $g(t)\in G$, see Marsden and Ratiu (1999) and Holm (2008). The simplest case is when $M= G$. This case, called Euler–Poincaré reduction, will be described in the present section.

In the Lagrangian framework, reduction by symmetry may be implemented in Hamilton’s principle via restricted variations in the reduced variational principle arising from variations on the corresponding Lie group. In the standard approach, for an arbitrary variation $\delta g$ of a curve $g(t)\in G$ in a Lie group *G*, the left-invariant reduced variables are $g^{-1}\dot{g}\in {\mathfrak {g}}$ in the Lie algebra ${\mathfrak {g}}= T_eG$. Their variations arise from variations on the Lie group and are given by

$$\begin{aligned} \delta \xi = \dot{\eta }+ \mathrm {ad}_\eta \xi \,, \end{aligned}$$

for $\eta := g^{-1}\delta g$. Here, the operation $\mathrm {ad}:{\mathfrak {g}}\times {\mathfrak {g}}\rightarrow {\mathfrak {g}}$ represents the adjoint action of the Lie algebra on itself via the Lie bracket, denoted equivalently as $\mathrm {ad}_\xi \eta = [\xi ,\eta ]$, and we will freely use either notation throughout the text. If the Lagrangian $L(g,\dot{g})$ is left-invariant under the action of *G*, the restricted variations $\delta \xi$ of the reduced Lagrangian $L(e,g^{-1}\dot{g})=:l(\xi )$ inherited from admissible variations of the solution curves on the group yield the Euler–Poincaré equation

2.2 $$\begin{aligned} \frac{\text{ d }}{\hbox {d}t}\frac{\partial l(\xi )}{\partial \xi } + \mathrm {ad}^*_\xi \frac{\partial l(\xi )}{\partial \xi }=0 \,. \end{aligned}$$

In this equation, $\mathrm{ad}^*: {\mathfrak {g}}\times {\mathfrak {g}}^*\rightarrow {\mathfrak {g}}^*$ is the dual of the adjoint Lie algebra action, ad. That is, $\langle \mathrm{ad}^*_\xi \mu ,\eta \rangle =\langle {\mu ,\mathrm ad}_\xi \eta \rangle$ for $\mu \in \mathfrak {g}^*$ and $\xi ,\eta \in \mathfrak {g}$, under the non-degenerate pairing $\langle \,\cdot \,,\, \cdot \,\rangle : {\mathfrak {g}}\times {\mathfrak {g}}^*\rightarrow \mathbb {R}$. Throughout this paper, we will restrict ourselves to semi-simple Lie algebras, so that the pairing is given by the Killing form, defined as

2.3 $$\begin{aligned} \kappa (\xi ,\eta ) := \mathrm {Tr}\left( \mathrm {ad}_\xi \mathrm {ad}_\eta \right) \, . \end{aligned}$$

In terms of the structure constants of the Lie algebra denoted as $c_{jk}^i$ for a basis $e_i,\,i=1,\dots ,\mathrm{dim}(\mathfrak {g})$, so that $[e_i,e_j]=c_{jk}^ie_i$, in which $\xi =\xi ^ie_i$ and $\eta =\eta ^je_j$, the Killing form takes the explicit form

$$\begin{aligned} \mathrm {Tr}(\mathrm {ad}_\xi \mathrm {ad}_\eta )= c_{im}^nc_{jn}^m \xi ^i \eta ^j\, . \end{aligned}$$

An important property of this pairing is its bi-invariance, written as

2.4 $$\begin{aligned} \kappa (\xi , \mathrm {ad}_\zeta \eta ) = \kappa (\mathrm {ad}_\xi \zeta ,\eta )\, , \end{aligned}$$

for every $\xi ,\eta ,\zeta \in \mathfrak {g}$. This pairing allows us to identify the Lie algebra with its dual, as the Killing form of semi-simple Lie algebras is non-degenerate. We will also use the property for compact Lie algebras, that the Killing form is negative definite and thus induces a norm on the Lie algebra, $\Vert \xi \Vert ^2:=-\kappa (\xi ,\xi )$. This function turns out to always be an invariant function on the coadjoint orbit, for every semi-simple Lie algebra; that is, it is a Casimir function. Indeed, an invariant function is in the kernel of the Lie–Poisson bracket $\{F,C\}(\mu ) := \kappa \left( \mu , \left[ \frac{\partial F}{\partial \mu },\frac{\partial C}{\partial \mu }\right] \right)$, $\forall F\in C({\mathfrak {g}}^*, \mathbb {R})$. For $C(\mu )= \frac{1}{2} \kappa (\mu ,\mu )$ it is straightforward to check using the bi-invariance (2.4) that this is true for any function *F*. In general, it is difficult to find other independent Casimir functions of semi-simple Lie algebra; see Perelomov and Popov (1968) and Thiffeault and Morrison (2000). Of course, the theory of semi-simple Lie algebras is standard and well developed, see for example Varadarajan (1984). However, for the sake of clarity, we will express the abstract notations of adjoint and coadjoint actions with respect to the Killing form. We may then identify ${\mathfrak {g}}^* \cong {\mathfrak {g}}$ for each semi-simple Lie algebra we treat here.

We now turn to the equivalent Clebsch formulation of the Euler–Poincaré equations via a constrained Hamilton’s principle, which we will use for implementing the noise in these systems. The Clebsch formulation of the Euler–Poincaré equation and its corresponding Lie–Poisson bracket on the Hamiltonian side has been explored extensively in ideal fluid dynamics (Holm and Kupershmidt 1983; Marsden and Weinstein 1983) and more recently in optimal control problems Gay-Balmaz and Ratiu (2011) and stochastic fluid dynamics Holm (2015). This earlier work should be consulted for detailed derivations of Clebsch formulations of Euler–Poincaré equations in the contexts of ideal fluids and optimal control problems. We will briefly sketch the Clebsch approach, as specialised to the applications treated here; since we will rely on it for the introduction of noise in finite-dimensional mechanical systems by following the approach of Holm (2015) for stochastic fluid dynamics. We first introduce the Clebsch variables $q\in {\mathfrak {g}}$ and $p\in {\mathfrak {g}}^*$, where *p* will be a Lagrange multiplier which enforces the dynamical evolution of *q* given by the Lie algebra action of $\xi \in {\mathfrak {g}}$, as $\dot{q}+ \mathrm {ad}_\xi q= 0$. Note the similarity of this equation with the constrained variations of the Lagrangian reduction theory. The Clebsch method in fluid dynamics (resp. optimal control) introduces auxiliary equations for advected quantities (resp. Lie algebra actions on state variables) as constraints in the Hamilton (resp. Hamilton-Pontryagin) variational principle $\delta S = 0$ with constrained action

2.5 $$\begin{aligned} S(\xi ,q,p) =\int l(\xi ) \hbox {d}t + \int \langle p, \dot{q} + \mathrm {ad}_\xi q \rangle \hbox {d}t\,. \end{aligned}$$

Taking free variations of *S* with respect to $\xi ,q$ and *p* yields a set of equations for these three variables which can be shown to be equivalent to the Euler–Poincaré equation (2.2). The relation between the Lie algebra vector $\xi \in {\mathfrak {g}}$ and the phase-space variables $(q,p)\in T_e^*G$ is given by the variation of the action *S* in (2.5) with respect to the velocity $\xi$ in (2.5). This variation yields the momentum map, $\mu : T_e^*G \rightarrow {\mathfrak {g}}^*$, given explicitly by

2.6 $$\begin{aligned} \mu := \frac{\partial l(\xi )}{\partial \xi } = \mathrm {ad}^*_qp\, . \end{aligned}$$

Unless specified otherwise, we will always use the notation $\mu$ for the conjugate variable to $\xi$. This version of the Clebsch theory is especially simple, as the Clebsch variables are also in the Lie algebra ${\mathfrak {g}}$. In general, it is enough for them to be in the cotangent bundle of a manifold $T^*M$ on which the group *G* acts by cotangent lifts. In this more general case, the adjoint and coadjoint actions must be replaced by their corresponding actions on $T^*M$, but the method remains the same. Another generalisation, which will be useful for us later, allows the Lagrangian to depend on both $\xi$ and *q*. In this case, the Euler–Poincaré equation will acquire additional terms depending on *q* and the Clebsch approach will be equivalent to semidirect product reduction (Holm et al. 1998). We will consider a simple case of this extension in Sect. 2.3 and in the treatment of the heavy top in Sect. 6.

### Structure Preserving Stochastic Deformations

We are now ready to deform the Euler–Poincaré equation (2.2) by introducing noise in the constrained Clebsch variational principle (2.5). In order to do this stochastic deformation, we introduce *n* independent Wiener processes $W_t^i$ indexed by $i=1,2,\dots ,n,$ and their associated stochastic potential fields $\Phi _i(q,p) \in \mathbb {R}$ which are prescribed functions of the Clebsch phase-space variables, (*q*, *p*). The stochastic processes used here are standard Weiner processes, as discussed, e.g., in Chen et al. (2015) and Ikeda and Watanabe (2014). The number of stochastic processes can be arbitrary, but we will assume it is equal to the dimension of the Lie algebra, $n=\mathrm{dim}({\mathfrak {g}})$. The constrained stochastic variational principle is then given by

2.7 $$\begin{aligned} S(\xi ,q,p) =\int l(\xi ) \hbox {d}t + \int \langle p,\circ dq + \mathrm {ad}_\xi q\, \hbox {d}t \rangle + \int \sum _{i=1}^n \Phi _i(q,p)\circ dW^i_t\, . \end{aligned}$$

In the stochastic action integral (2.7) and hereafter, the multiplication symbol $\circ$ denotes a stochastic integral in the Stratonovich sense (Kloeden and Platen 1991). The Stratonovich formulation is the only choice of stochastic integral that admits the classical rules of calculus (e.g., integration by parts, the change of variables formula, etc.). Therefore, the Stratonovich formulation is indispensable in variational calculus and optimal control. The free variations of the action functional (2.7) may now be taken, and they will yield stochastic processes for the three variables $\xi ,q$ and *p*.

For convenience in the next step of deriving a stochastic Euler–Poincaré equation, we will assume that the Lagrangian $l(\xi )$ in the action (2.7) is hyperregular, so that $\xi$ may be obtained from the fibre derivative $\frac{\partial l(\xi )}{\partial \xi } = \mathrm {ad}^*_qp$. We will also specify that the stochastic potentials $\Phi _i(q,p)$ should depend only on the momentum map $\mu = \mathrm {ad}^*_q p$, so that the resulting stochastic equation will be independent of *q* and *p*. Following the detailed calculations in Holm (2015), we then find the stochastic Euler–Poincaré equation

2.8 $$\begin{aligned} d \frac{\partial l(\xi )}{\partial \xi } + \mathrm {ad}^*_\xi \frac{\partial l(\xi )}{\partial \xi } \hbox {d}t - \sum _i \mathrm {ad}^*_\frac{\partial \Phi _i(\mu )}{\partial \mu } \frac{\partial l(\xi )}{\partial \xi }\circ dW_t^i=0\,. \end{aligned}$$

In terms of the stochastic process

2.9 $$\begin{aligned} dX= \xi \hbox {d}t - \sum _i \frac{\partial \Phi _i(\mu )}{\partial \mu } \circ dW_t^i \,,\quad \hbox {with}\quad \mu =\frac{\partial l(\xi )}{\partial \xi }\, , \end{aligned}$$

the stochastic Euler–Poincaré equation (2.8) may be expressed in compact form, as

2.10 $$\begin{aligned} d\mu + \mathrm {ad}^*_{dX} \mu =0\,. \end{aligned}$$

The introduction of noise in the Clebsch-constrained variational principle rather than using reduction theory provides a transparent approach for dealing with stochastic processes on Lie groups and constrained variations arising for such processes. In this approach, the momentum map plays the same central role in both the deterministic and stochastic formulations. See Arnaudon et al. (2014) for a different approach, resulting in the derivation and analysis of deterministic expectation-value Euler–Poincaré equations using reduction by symmetry with conditional expectation.

#### Remark 2.1

(Reduction with noise) The stochastic Euler–Poincaré equation (2.8) arises from a stochastic reduction by symmetry, as follows. First, the reconstruction relation $\dot{g}= g \xi$ in the deterministic case has its stochastic counterpart

2.11 $$\begin{aligned} dg= g \xi \hbox {d}t + \sum _i g\sigma _i \circ dW_t^i\, , \end{aligned}$$

where $\sigma _i:= -\,\frac{\partial \Phi _i(\mu )}{\partial \mu }$, and the expressions $g \xi$ and $g \sigma _i$ are understood as the tangent of the left action of the group on itself; or equivalently, the left action of the group on its Lie algebra. Equation (2.8) then results from taking the variation of $g^{-1} dg$ with (2.11) and comparing with the derivative of $g^{-1}\delta g$ while setting $d(\delta g)= \delta (dg)$. This gives the variation $\delta \xi$ as

2.12 $$\begin{aligned} \delta \xi = d\eta + \mathrm {ad}_{(g^{-1} dg)}\eta = d\eta + \mathrm {ad}_\xi \eta \, \hbox {d}t + \sum _i \mathrm {ad}_{\sigma _i}\eta \circ dW^i_t\, , \end{aligned}$$

where $\eta = g^{-1}\delta g\in {\mathfrak {g}}$ is arbitrary, except that $\delta g(0)=0=\delta g(T)$ at the endpoints in time $t\in [0,T]$. Then, using these constrained variations in the reduced variational principle $\delta \int l(\xi )\hbox {d}t = 0$ yields the stochastic Euler–Poincaré equation (2.8), by the following calculation,

2.13 $$\begin{aligned} \begin{aligned} 0&= \delta \int l(\xi )\hbox {d}t = \int \left\langle \frac{\delta l}{\delta \xi } \,,\, \delta \xi \right\rangle \hbox {d}t = \int \left\langle \frac{\delta l}{\delta \xi } \,,\, d\eta + \mathrm {ad}_{(g^{-1} dg)}\eta \right\rangle \hbox {d}t\\&= \int \left\langle -\,d\frac{\delta l}{\delta \xi } + \mathrm {ad}^*_{(g^{-1} dg)} \frac{\delta l}{\delta \xi } \,,\, \eta \right\rangle \hbox {d}t + \left\langle \frac{\delta l}{\delta \xi } \,,\, \eta \right\rangle \bigg |_0^T \end{aligned} \end{aligned}$$

upon imposing the condition that $\eta$ vanishes at the endpoints in time.

As in the deterministic case, various generalisations of this theory are possible. For example, as mentioned earlier, the Clebsch phase-space variables can also be defined in $T^*M$, and the Lagrangian can depend on *q* for systems of semidirect product type (Gay-Balmaz and Holm 2017). Another generalisation is to let the stochastic potentials $\Phi _i(\mu )$ also depend separately on *q* in the semidirect product setting, as we will see later.

After having defined the Stratonovich stochastic process (2.8), one may compute its corresponding Itô form, which is readily given in terms of the $\mathrm {ad}^*$ operation by

2.14 $$\begin{aligned} \begin{aligned} d \frac{\partial l(\xi )}{\partial \xi } + \mathrm {ad}^*_\xi \frac{\partial l(\xi )}{\partial \xi } \hbox {d}t&+ \sum _i \mathrm {ad}^*_{\sigma _i} \frac{\partial l(\xi )}{\partial \xi }dW_t^i - \frac{1}{2} \sum _i \mathrm {ad}^*_{\sigma _i}\left( \mathrm {ad}^*_{\sigma _i}\frac{\partial l(\xi )}{\partial \xi }\right) \hbox {d}t=0\, , \end{aligned} \end{aligned}$$

where $\sigma _i:= -\,\frac{\partial \Phi _i(\mu )}{\partial \mu }$. Notice that the indices for $\sigma _i$ in the Itô sum in (2.14) are the same, and the $\sigma _i$ may be taken as a basis of the underlying vector space. In terms of $\mu := \frac{\partial l(\xi )}{\partial \xi }$ the Itô stochastic Euler–Poincaré equation (2.14) may be expressed equivalently as

2.15 $$\begin{aligned} d \mu + \mathrm {ad}^*_\xi \mu \hbox {d}t + \sum _i \mathrm {ad}^*_{\sigma _i} \mu dW_t^i - \frac{1}{2} \sum _i \mathrm {ad}^*_{\sigma _i}\left( \mathrm {ad}^*_{\sigma _i}\mu \right) \hbox {d}t=0\,. \end{aligned}$$

Another formulation of the stochastic Euler–Poincaré equation in (2.8) which will be used later in deriving the Fokker–Planck equation is the stochastic Lie–Poisson equation

2.16 $$\begin{aligned} d f(\mu )&= \left\langle \mu , \left[ \frac{\partial f}{\partial \mu }, \frac{\partial h}{\partial \mu }\right] \right\rangle \hbox {d}t + \sum _i\left\langle \mu , \left[ \frac{\partial f}{\partial \mu }, \frac{\partial \Phi _i}{\partial \mu }\right] \right\rangle \circ dW_t^i \end{aligned}$$

2.17 $$\begin{aligned}&=: \{f,h\} \hbox {d}t +\sum _i \{ f,\Phi _i\} \circ dW_i\,, \end{aligned}$$

where the Lie–Poisson bracket $\{\cdot ,\cdot \}$ is defined just as in the deterministic case, from the adjoint action and the pairing on the Lie algebra ${\mathfrak {g}}$.

### Extension to Semidirect Product Systems

As discussed in Holm et al. (1998), “It turns out that semidirect products occur under rather general circumstances when the symmetry in $T^*G$ is broken”. The geometric mechanism for this remarkable fact is that under reduction by symmetry, a semidirect product of groups emerges whenever the symmetry in the phase space is broken. The symmetry breaking produces new dynamical variables, living in the coset space formed from taking the quotient $G/G_a$ of the original unbroken symmetry *G* by the remaining symmetry $G_a$ under the isotropy subgroup of the new variables. These new dynamical variables form a vector space $G/G_a\simeq V$ on which the unbroken symmetry acts as a semidirect product, $G\,\circledS \,V$. In physics, elements of the vector space *V* corresponding to the new variables are called “order parameters”. Typically, in physics, the original symmetry is broken by the introduction of potential energy depending on variables which reduce the symmetry to the isotropy subgroup of the new variables. Dynamics on the semidirect product $G\,\circledS \,V$ results, and what had previously been flowed under the action of the unbroken symmetry now becomes flow plus waves, or oscillations, produced by the exchange of energy between its kinetic and potential forms. The heavy top is the basic example of this phenomenon, and it will be treated in Sect. 6. The semidirect product motion for the heavy top arises in the presence of gravity when the support point of a freely rotating rigid body is shifted away from its centre of mass.

With this connection between symmetry breaking and semidirect products in mind, we now extend the stochastic Euler–Poincaré equations to include semidirect product systems. We refer to Holm et al. (1998) for a complete study of these systems. Although the deterministic equations of motion in Holm et al. (1998) are derived from reduction by symmetry, we will instead incorporate noise by simply extending the Clebsch-constrained variational principle used in the previous section.

The generalisation proceeds, as follows. We will begin by assuming that the Clebsch phase-space variables comprise the elements of $T^*V$ for a given vector space *V* on which the Lie group *G* acts freely and properly. In fact we will have $(q,p)\in V\times V^*$. However, in this work, we will restrict ourselves to the case where *V* is the underlying vector space of ${\mathfrak {g}}$. Following the notation of Ratiu (1981), we denote $\overline{ {\mathfrak {g}}}= V$ in the sequel. Then, from the Killing form on ${\mathfrak {g}}$, denoted by $\kappa :{\mathfrak {g}}\times {\mathfrak {g}} \rightarrow \mathbb {R}$, there is a bi-invariant extension of the Killing form on ${\mathfrak {g}}\circledS \overline{{\mathfrak {g}} }$ defined as

2.18 $$\begin{aligned} \kappa _s \left( ( \xi _1, \xi _2) , (\eta _1, \eta _2) \right) := \kappa ( \xi _1,\eta _2) + \kappa ( \xi _2, \eta _1)\, . \end{aligned}$$

Although this pairing is non-degenerate and bi-invariant, we will not use it for the definition of the dual of the semidirect algebra ${\mathfrak {g}}\circledS V$. Instead, we will use the sum of both Killing forms, namely

2.19 $$\begin{aligned} \kappa _0 \left( ( \xi _1, \xi _2) , (\eta _1, \eta _2) \right) := \kappa ( \xi _1,\eta _1) + \kappa ( \xi _2, \eta _2)\, . \end{aligned}$$

The group action is defined via the adjoint representation of *G* on $V=\overline{{\mathfrak {g}}}$, given by $(g_1,\eta _1)(g_2,\eta _2) = (g_1g_2, \eta _1 + \mathrm {Ad}_{g_1}\eta _2)$. We then directly have the infinitesimal adjoint and coadjoint actions as

2.20 $$\begin{aligned} \begin{aligned} \mathrm {ad}_{(\xi _1,q_1)} (\xi _2,q_2)&= ( \mathrm {ad}_{\xi _1}\xi _2 , \mathrm {ad}_{\xi _1}q_2+\mathrm {ad}_{q_1}\xi _2)\,,\\ \mathrm {ad}^*_{(\xi ,q)} (\mu ,p)&= ( \mathrm {ad}^*_\xi \mu + \mathrm {ad}^*_qp, \mathrm {ad}^*_\xi p)\, , \end{aligned} \end{aligned}$$

where the coadjoint action is taken with respect to $\kappa _0$ in (2.19). The extended Killing form $\kappa _s$ defined in (2.18), gives, apart from $\kappa (\eta ,\eta )$ with $\eta \in \overline{{\mathfrak {g}}}$, a second invariant function on the coadjoint orbit

$$\begin{aligned} \kappa _s\left( (\xi ,\eta ), (\xi ,\eta )\right) = 2\kappa (\xi , \eta )\, , \end{aligned}$$

found from the bi-invariance of the Killing form $\kappa _s$. One then replaces the corresponding Lie algebra actions in the Clebsch-constrained variational principle (2.7), to obtain the stochastic process with semidirect product

2.21 $$\begin{aligned} \begin{aligned} d \left( \mu , q \right)&+ \mathrm {ad}^*_{(\xi ,r)}\left( \mu , q\right) \hbox {d}t + \sum _i \mathrm {ad}^*_{\left( \frac{\partial \Phi _i(\mu ,q)}{\partial \mu },\frac{\partial \Phi _i(\mu ,q)}{\partial q}\right) } \left( \mu , q \right) \circ dW_t^i=0\, , \end{aligned} \end{aligned}$$

where $$l:{\mathfrak {g}}\circledS V\rightarrow \mathbb {R} $$, $$\Phi _i:{\mathfrak {g}}^*\rightarrow \mathbb {R}$$,

2.22 $$\begin{aligned} \frac{\partial l(\xi ,q)}{\partial \xi }=: \mu \qquad \mathrm {and}\qquad \frac{\partial l(\xi ,q)}{\partial q}=: r\,. \end{aligned}$$

#### Remark 2.2

(*q* dependence on the stochastic potentials) Notice that the stochastic potentials do not depend on the advected variables. This is a consequence of the Clebsch construction. By using the Hamilton-Pontryagin derivation of this equation, the potentials may depend on the advected variables, see the remark 3.2 or Gay-Balmaz and Holm (2017) for more details. We will write here the dependence on the advected variables for completeness, but we will not use it in the examples.

After taking the Legendre transform of reduced Lagrangian *l*, we have the Hamiltonian derivatives

2.23 $$\begin{aligned} \frac{\partial h(\mu ,q)}{\partial \mu }=: \xi \qquad \mathrm {and}\qquad \frac{\partial h(\mu ,q)}{\partial q}=: -\,r\,, \end{aligned}$$

for $h:{\mathfrak {g}}^*\circledS V^*\rightarrow \mathbb {R}$. By substituting into (2.21) the expressions in (2.20) for the coadjoint action, we obtain the system

2.24 $$\begin{aligned} \begin{aligned}&d \mu + \left( \mathrm {ad}^*_\xi \mu + \mathrm {ad}^*_r q\right) {\text{ d }}t + \sum _i \left( \mathrm {ad}^*_\frac{\partial \Phi _i(\mu ,q)}{\partial \mu } \mu + \mathrm {ad}^*_\frac{\partial \Phi _i(\mu ,q)}{\partial q} q \right) \circ dW_t^i =0 \,,\\&\quad d q + \mathrm {ad}^*_\xi q {\text{ d }}t + \sum _i \mathrm {ad}^*_\frac{\partial \Phi _i(\mu ,q)}{\partial \mu }q \circ dW_t^i=0\,. \end{aligned} \end{aligned}$$

Although the number of stochastic potentials $\Phi _i$ which one may consider is arbitrary, we shall find it convenient for our purposes to restrict to a maximum of $n= \mathrm {dim}({\mathfrak {g}}) + \mathrm {dim}(V)$ such potentials. In fact, the potentials associated with *V* will not be fully treated here.

#### Remark 2.3

General setting The semidirect product theory we have described here is the simplest instance of it, as we are using a particular vector space *V*. In general, the advected quantities can also be in a Lie algebra, or an arbitrary manifold, provided the action of the group *G* on it is free and proper.

### The Fokker–Planck Equation and Stationary Distributions

We derive here a geometric version of the classical Fokker–Planck equation (or forward Kolmogorov equation) using our SDE (2.8). Recall that the Fokker–Planck equation describes the time evolution of the probability distribution $\mathbb {P}$ for the process driven by (2.8). We refer to Ikeda and Watanabe (2014) for the standard textbook treatment of stochastic processes. Here, we will consider $\mathbb {P}$ as a function $C({\mathfrak {g}}^*)$ with the additional property that $\int _\mathfrak {g^*} \mathbb {P} d\mu = 1$. First, the generator of the process (2.8) can be readily found from the Lie–Poisson form (2.14) of the stochastic process (2.17) to be

2.25 $$\begin{aligned} Lf(\mu ) = \left\langle \mathrm {ad}^*_\xi \mu ,\frac{\partial f}{\partial \mu }\right\rangle - \sum _i\left\langle \mathrm {ad}^*_{\sigma _i}\mu ,\frac{\partial }{\partial \mu } \left\langle \mathrm {ad}^*_{\sigma _i}\mu ,\frac{\partial f}{\partial \mu }\right\rangle \right\rangle \, , \end{aligned}$$

where $\langle \,\cdot \,,\, \cdot \,\rangle$ still denotes the Killing form on the Lie algebra ${\mathfrak {g}}$ and $f\in C({\mathfrak {g}}^*$) is an arbitrary function of $\mu$. The Fokker–Planck equation

$$\begin{aligned} \partial _t \mathbb {P}= L^*\mathbb {P} \end{aligned}$$

obtained from the formula (2.25) is proved in the following proposition, cf. Holm et al. (2016).

#### Proposition 2.4

The forward Kolmogorov operator is

2.26 $$\begin{aligned} L^*\mathbb {P}(\mu ) = -\{ h,\mathbb {P}\} - \frac{1}{2} \sum _i\{\Phi _i, \{\Phi _i,\mathbb {P}\}\} \,. \end{aligned}$$

#### Proof

We first consider the drift term of (2.25) written in Lie-Bracket form and use the Leibniz property of the Lie–Poisson bracket to get

2.27 $$\begin{aligned} \int \mathbb {P} \{ h,f\} d\mu = - \int f\{h, \mathbb {P}\} d\mu + \int \{h,f\mathbb {P}\}d\mu \, . \end{aligned}$$

Recall that a non-degenerate Poisson bracket can be expressed in term of a symplectic form as $\{F,G\}= \omega (X_F,X_G)$ where $X_F$ and $X_G$ are the Hamiltonian vector fields associated with the function *F* and *G*. Now, the symplectic form on the coadjoint orbit is an exact 2-form, that is $\omega = d\theta$ for some 1-form $\theta$. These facts mean that provided that we have vanishing or periodic boundary conditions on the coadjoint orbits, the integration of the Lie–Poisson bracket corresponds to the integration of the exterior differential of a 1-form. This integral vanishes by using Stokes theorem and suitable boundary conditions. For the double bracket term, we similarly obtain

$$\begin{aligned} \int \mathbb {P} \{\Phi _i, \{ \Phi _i,f\}\} d\mu&= - \int \{\Phi _i,f\}\{\Phi _i, \mathbb {P}\} d\mu + \int \{\Phi _i ,\{\Phi _i,f\}\mathbb {P}\}d\mu \\&= \int f\{\Phi _i,\{\Phi _i, \mathbb {P}\}\} d\mu - \int \{\Phi _i,f\{\Phi _i, \mathbb {P}\}\} d\mu \\&\quad +\, \int \{\Phi _i ,\{\Phi _i,f\}\mathbb {P}\}d\mu \, , \end{aligned}$$

where the last two terms vanishes from the same argument as before, that is using integration by parts. Collecting terms give the forward Kolmogorov operator (2.26). $\square$

In (2.26), we recover the Lie–Poisson formulation (2.17) of the Euler–Poincaré equation together with a dissipative term arising from the noise of the original SDE in a double Lie–Poisson bracket form. This formulation gives the following theorem for stationary distributions of (2.25):

#### Theorem 2.5

The stationary distribution $\mathbb {P}_\infty$ of the Fokker–Planck equation (2.25), i.e., $L^*\mathbb {P}_\infty =0$ is uniform on the coadjoint orbits on which the SDE (2.8) evolves.

#### Proof

By a standard result in functional analysis, see for example (Villani 2009), a linear differential operator of the form $L = B + \frac{1}{2} \sum _i A_i^2$ has the property that $\mathrm {ker}(L) = \mathrm {ker}(A_i)\cap \mathrm {ker}(B)$, where here $A_i = \{\Phi _i, \cdot \}$ and $B = \{h, \cdot \}$. Consequently, for every smooth function *f*, the only functions *g* which satisfy $\{ f,g \}= 0$ are the Casimirs, or invariant functions, on the coadjoint orbits. When restricted to a coadjoint orbit, these functions become constants. Hence, the stationary distribution $\mathbb {P}_\infty$ is a constant on the coadjoint orbit identified by the initial conditions of the system. $\square$

Since the dynamics are restricted to the coadjoint orbits, for the probability distribution $\mathbb {P}$ to tend to a constant, yet remain normalisable, satisfying $\int _{{\mathfrak {g}}^*} \mathbb {P}(\mu )d\mu = 1$, the value of the density must tend to the inverse of the volume of the coadjoint orbit. Of course, the compactness of the coadjoint orbit is equivalent to $\mathbb {P}_\infty >0$. For non-compact orbits, Theorem 2.5 is still valid, and it will imply an asymptotically vanishing stationary distribution, in the same sense as for the stationary solution of the heat equation on the real line. In this case, a more detailed analysis of the stationary distribution can be performed by studying marginals, or projections onto a compact subspace of the coadjoint orbit.

Examples of non-compact coadjoint orbits arise in the semidirect product setting. First, the Fokker–Planck equation for the semidirect product stochastic process (2.21) is given by

2.28 $$\begin{aligned} \begin{aligned} Lf(\mu ,q)&= \left\langle \mathrm {ad}^*_{(\xi ,r)} (\mu ,q) ,\left( \frac{\partial f(\mu ,q)}{\partial \mu }, \frac{\partial f(\mu ,q)}{\partial q}\right) \right\rangle - \\&\quad -\, \sum _i\left\langle \mathrm {ad}^*_{(\sigma _i,\eta _i)}(\mu ,q), \left\{ \frac{\partial }{\partial \mu } \left\langle \mathrm {ad}^*_{(\sigma _i,\eta _i)}(\mu ,q) ,\left( \frac{\partial f(\mu ,q)}{\partial \mu },\frac{\partial f(\mu ,q)}{\partial q}\right) \right\rangle \right. \right. , \\&\quad \times \left. \left. \frac{\partial }{\partial q} \left\langle \mathrm {ad}^*_{(\sigma _i,\eta _i)}(\mu ,q) ,\left( \frac{\partial f(\mu ,q)}{\partial \mu },\frac{\partial f(\mu ,q)}{\partial q}\right) \right\rangle \right\} \right\rangle , \end{aligned} \end{aligned}$$

where $\sigma _i :=- {\partial \Phi _i}/{\partial \mu }$ and $\eta _i := -{\partial \Phi _i}/{\partial q}$. The pairing used here is the sum of the pairings on ${\mathfrak {g}}$ and on *V*, given by $\kappa _0$ in (2.19). Note that for some values of index *i*, the vector fields $\sigma _i$ or $\eta _i$ may be absent. One can check that $L^*= L$; so that *L* generates the Lie–Poisson Fokker–Planck equation for the probability density $\mathbb {P}(\mu ,q)$. As before, upon using the explicit form of the coadjoint actions, one finds

2.29 $$\begin{aligned} \begin{aligned} Lf(\mu ,q)&= \left\langle \mathrm {ad}^*_\xi \mu + \mathrm {ad}^*_q r , \frac{\partial f}{\partial \mu } \right\rangle + \left\langle \mathrm {ad}^*_\xi q ,\frac{\partial f}{\partial q}\right\rangle -\\&\quad -\, \sum _i\left\langle \mathrm {ad}^*_{\sigma _i} \mu + \mathrm {ad}^*_q \eta _i , \frac{\partial A_i}{\partial \mu } \right\rangle -\sum _i \left\langle \mathrm {ad}^*_{\sigma _i} q ,\frac{\partial A_i}{\partial q}\right\rangle , \\ \mathrm {where}\qquad A_i:&= \left\langle \mathrm {ad}^*_{\sigma _i} \mu + \mathrm {ad}^*_q \eta _i , \frac{\partial f}{\partial \mu } \right\rangle + \left\langle \mathrm {ad}^*_{ \sigma _i} q ,\frac{\partial f}{\partial q}\right\rangle \, . \end{aligned} \end{aligned}$$

The Fokker–Planck equation (2.28) provides a direct corollary of Theorem 2.5.

#### Corollary 2.5.1

The stationary distribution $\mathbb {P}_\infty (\mu ,q)$ of (2.28) is constant on the coadjoint orbit corresponding to the initial conditions of the stochastic process (2.21).

As mentioned earlier, the coadjoint orbit of this system is not compact, even if it had been compact for the Lie algebra ${\mathfrak {g}}$. Nevertheless, we can study the marginal distributions

2.30 $$\begin{aligned} \mathbb {P}^1(\mu )&:= \int \mathbb {P}(\mu ,q)dq\qquad \mathrm { and} \end{aligned}$$

2.31 $$\begin{aligned} \mathbb {P}^2(q)&:= \int \mathbb {P}(\mu ,q)d\mu \, , \end{aligned}$$

which of course extend to stationary marginal distributions $\mathbb {P}^1_\infty$ and $\mathbb {P}^2_\infty$. With these marginal distributions, we can get more information about the stationary distribution of the semidirect product Lie–Poisson Fokker–Planck equation (2.28), as summarised in the next theorem.

#### Theorem 2.6

For a semi-simple Lie algebra ${\mathfrak {g}}$ and $V= \overline{{\mathfrak {g}}}$, the marginal stationary distributions defined in (2.30) and (2.31) of the Fokker–Planck equation (2.28), with $\eta _i=0$, for all *i*, have the following forms.

1. The stationary distribution $\mathbb {P}^2_\infty (q)$ is constant on the *q*-dependent subspace of the coadjoint orbit. If the Lie algebra ${\mathfrak {g}}$ is non-compact, the constant is zero.
2. The stationary distribution $\mathbb {P}^1_\infty (\mu )$ restricted to $\kappa (\mu ,\mu )$ is constant.
3. If ${\mathfrak {g}}$ is compact, the stationary distribution $\mathbb {P}^1_\infty (\mu )$ is linearly bounded in time in the direction perpendicular to $\kappa (\mu ,\mu )$.

#### Proof

We will compute the stationary marginal distributions separately, but first recall that the stationary distribution $\mathbb {P}(\mu ,q)$ is constant on the Casimir level sets given by the initial conditions.

(1) By integrating the Fokker–Planck equation (2.28) over $\mu$, one obtains

2.32 $$\begin{aligned} L\mathbb {P}^2(q)&= \int \left\langle \mathrm {ad}^*_\xi q ,\frac{\partial \mathbb {P}(\mu ,q)}{\partial q}\right\rangle d\mu -\frac{1}{2} \left\langle \mathrm {ad}^*_{ \sigma _i} q ,\frac{\partial }{\partial q} \left\langle \mathrm {ad}^*_{\sigma _i} q ,\frac{\partial \mathbb {P}^2(q)}{\partial q}\right\rangle \right\rangle \, , \end{aligned}$$

where we have used the property that the coadjoint action is divergence-free (because of the anti-symmetry of the adjoint action, when identified with the coadjoint action via the Killing form) and have recalled that the Lie algebra is either compact, or $\mathbb {P}(\mu ,q)= 0$ for the boundary conditions.

Only the advection term remains in (2.32), as $\xi =\frac{\partial h}{\partial \mu }$ depends on $\mu$. Nevertheless, an argument similar to that for the proof of Theorem 2.5 may be applied here to give the result of constant marginal distribution on the *q* dependent part of the coadjoint orbits. Again, if the Lie algebra is non-compact, then the probability density $\mathbb {P}^2_\infty (q)$ must vanish because of the normalisation.

(2) We first integrate the Fokker–Planck equation (2.28) with respect to the *q* variable to find

2.33 $$\begin{aligned} L\mathbb {P}^1(\mu )&= \left\langle \mathrm {ad}^*_\xi \mu , \frac{\partial \mathbb {P}^1}{\partial \mu } \right\rangle - \frac{1}{2} \sum _i\left\langle \mathrm {ad}^*_{\sigma _i} \mu , \frac{\partial }{\partial \mu } \left\langle \mathrm {ad}^*_{\sigma _i} \mu , \frac{\partial \mathbb {P}^1}{\partial \mu } \right\rangle \right\rangle \, , \end{aligned}$$

where we have again used that the coadjoint action is divergence-free, the same boundary conditions and the fact that $\langle \mathrm {ad}_q \xi , \frac{\partial \mathbb {P}}{\partial \mu }\rangle = 0,\, \forall \xi$ since $\frac{\partial \mathbb {P}}{\partial \mu }$ is aligned with *q*. Indeed, $\mathbb {P}$ is a function of the Casimirs, and thus is a function of $\kappa _s((\mu ,q),(\mu ,q))$. This fact prevents us from directly invoking Theorem 2.5 as we would find that $\mathbb {P}^1$ is indeed constant on $\kappa (\mu ,\mu )$, but $\mu$ does not have an invariant norm. Nevertheless, we can still use this theorem by restricting $\mathbb {P}^1$ to the sphere $\kappa (\mu ,\mu )$, or equivalently simply considering polar coordinates for $\mu$ and discarding the radial coordinate. In this case, we can invoke Theorem 2.5 and obtain the result of a constant marginal distribution $\mathbb {P}^1_\infty$ projected on the coadjoint orbit of the Lie algebra ${\mathfrak {g}}$ alone.

(3) We compute the time derivative of the quantity $\Vert \mu \Vert ^2:= -\,\kappa (\mu ,\mu )$, which is positive definite and thus defines a norm, to get an upper estimate of the form

$$\begin{aligned} \frac{\text{ d }}{{\text{ d }}t}\frac{1}{2} \Vert \mu \Vert ^2&= \langle \mu , \dot{\mu }\rangle = \langle \mathrm {ad}_r q, \mu \rangle \le \Vert r\Vert \Vert q\Vert \Vert \mu \Vert \, . \end{aligned}$$

Then, because $\Vert q\Vert =\sqrt{-\kappa (q,q)}$ is constant, and provided that *r* is bounded, we can integrate to find

2.34 $$\begin{aligned} \Vert \mu (t)\Vert \le \Vert \mu (0)\Vert +\alpha t\, , \end{aligned}$$

where $\alpha$ is a constant depending on the Lie algebra and the Hamiltonian. $\square$

#### Remark 2.7

(On ergodicity) An important question about any given dynamical system is whether its solution is ergodic. This question needs some clarification for the systems considered here. First, notice that the deterministic systems are not ergodic, as they are Hamiltonian systems with extra conserved quantities given by the Casimirs. If the $\sigma _i$ span the entire Lie algebra, there is a constant invariant measure on the level set of the Casimir given by the initial conditions. This means that we have the ergodicity property on the coadjoint orbits but not on the full Euclidian space in which the coadjoint orbits are embedded. The ergodicity must then only be defined with respect to the coadjoint orbit, or the system will not be seen to be ergodic. Finally, the cases where the $\sigma _i$ do not span the Lie algebra must be treated individually, depending on the system in hand. For example with the rigid body in Sect. 5, having two independent non-trivial $\sigma _i$ is sufficient for ergodicity, while having only one $\sigma _i$ will make the system integrable, and thus non-ergodic on the coadjoint orbit, or momentum sphere.

*Summary* This section has reviewed the framework for the study of noise in dynamical systems defined on coadjoint orbits and has illustrated how noise may be included in these systems, so as to preserve the deterministic coadjoint orbits. This preservation property is seen clearly in the Clebsch formulation because the deterministic and stochastic systems share the same momentum map, whose level sets define the coadjoint orbits. The systems we have considered are the Euler–Poincaré equations on semi-simple finite-dimensional Lie groups and the semidirect product structures which appear when the advected quantities are introduced into the underlying vector space of the Lie algebra of the Lie group. These structures are not the most general. However, their study has allowed us to use the properties of the natural pairing given by the Killing form to prove a few illustrative results in a simple and transparent way. In particular, we showed that the stationary solution of the Fokker–Planck equation, written in Lie–Poisson form, is constant on the coadjoint orbits. In the semidirect product setting, a bit more care was needed to obtain similar results for the marginal distributions, as the coadjoint orbits are not compact in this case. We will illustrate our approach with the two basic examples of the rigid body and heavy top in Sects. 5 and 6, where more will be said about these systems, and in particular about their integrability.

## Dissipation and Invariant Measures

In the previous section, we described a structure preserving stochastic deformation of mechanical systems with symmetries. The preserved structure is the coadjoint orbit of the deterministic system. Namely, the stochastic process still belongs to one of these orbits, characterised by the initial conditions of the system. This preservation is reflected in the strict conservation of particular integrals of motion, called Casimirs. In general, these are the only conserved quantities of our stochastic processes. Indeed, the energy is not conserved, apart from very particular choices of the energy and the stochastic potentials as we will see for some examples. The energy is not strictly decaying either but is subject to random fluctuations. The complexity of the energy evolution has hindered us from studying it in full generality in the previous sections. In the present section, however, we will investigate the energy behaviour for particular mechanical examples subject to dissipation and random fluctuations. The type of energy dissipation that we will introduce in Sect. 3.1 also preserves the coadjoint orbits. Consequently, the dissipation is compatible with our stochastic deformation. The main outcome after introducing this dissipation is the emergence of a balance between noise and dissipation which will make the stationary solution of the Fokker–Planck equation energy dependent, as we will see in Sect. 3.2 and in the proof of existence of random attractors in Sect. 4.

### Double Bracket Dissipation

To augment the stochastic processes introduced in the previous section, we will add a type of dissipation for which the solutions of the stochastic process will still lie on the deterministic coadjoint orbit. For this purpose, we will use double bracket dissipation, which was studied in detail in Bloch et al. (1996) and was generalised recently in Gay-Balmaz and Holm (2013) and Gay-Balmaz and Holm (2014). We will follow the latter works to include an energy dissipation which preserves the Casimir functions. We will not review this theory in detail here. Instead, we refer the reader to Gay-Balmaz and Holm (2013) for a detailed discussion of Euler–Poincaré selective decay dissipation and Gay-Balmaz and Holm (2014) for the semidirect product extension.

For the stochastic process (2.10), the dissipative stochastic Euler–Poincaré equation written in Hamiltonian form is

3.1 $$\begin{aligned} d\mu + \mathrm {ad}^*_\frac{\partial h}{\partial \mu } \mu \, {\text{ d }}t + \theta \, \mathrm {ad}^*_\frac{\partial C}{\partial \mu } \left[ \frac{\partial C}{\partial \mu }, \frac{\partial h}{\partial \mu } \right] ^\flat {\text{ d }}t + \sum _i\mathrm {ad}^*_{\sigma _i} \mu \circ dW_t^i = 0 \,, \end{aligned}$$

where $\theta >0$ parametrises the rate of energy dissipation and *C* is a chosen Casimir of the coadjoint orbit. For convenience, we are using the isomorphism $\flat :{\mathfrak {g}}\rightarrow {\mathfrak {g}}^*$ defined via the Killing form of ${\mathfrak {g}}$. The converse isomorphism will be denoted $\sharp :{\mathfrak {g}}^*\rightarrow {\mathfrak {g}}$. The corresponding generalisation of selective decay for the semidirect product stochastic process (2.21), following (Gay-Balmaz and Holm 2014), is given by

3.2 $$\begin{aligned} \begin{aligned}&d(\mu ,q) + \mathrm {ad}^*_{(\xi ,r)} ( \mu ,q)\, {\text{ d }}t + \theta \, \mathrm {ad}^*_{\left( \frac{\partial C}{\partial \mu },\frac{\partial C}{\partial q}\right) } \left[ \left( \frac{\partial C}{\partial \mu },\frac{\partial C}{\partial q}\right) , ( \xi ,r) \right] ^\flat {\text{ d }}t \\&\quad + \sum _i\mathrm {ad}^*_{(\sigma _i,\eta _i)} (\mu ,q) \circ dW_t^i = 0\, , \end{aligned} \end{aligned}$$

where $\xi = \frac{\partial h}{\partial \mu }$, and the quantities *h* and *r* are defined in equation (2.23). Equation (3.2) may be written equivalently as a system of equations, by using the actions given in (2.20). Namely,

3.3 $$\begin{aligned} \begin{aligned}&d\mu + (\mathrm {ad}^*_\xi \mu +\mathrm {ad}^*_r q)\, {\text{ d }}t + \theta \, \mathrm {ad}^*_{\frac{\partial C}{\partial \mu }} \left[ \frac{\partial C}{\partial \mu }, \xi \right] ^\flat {\text{ d }}t \,+\, \theta \, \mathrm {ad}^*_\frac{\partial C}{\partial q} \left( \mathrm {ad}_\frac{\partial C}{\partial \mu } r + \mathrm {ad}_\frac{\partial C}{\partial q} \xi \right) ^\flat {\text{ d }}t \\&\quad +\, \sum _i \left( \mathrm {ad}^*_{\sigma _i}\mu +\mathrm {ad}^*_{\eta _i}q\right) \circ dW_t^i = 0,\\&\quad dq + \mathrm {ad}^*_\xi q\, {\text{ d }}t + \theta \, \mathrm {ad}^*_\frac{\partial C}{\partial \mu } \left( \mathrm {ad}_\frac{\partial C}{\partial \mu } r- \mathrm {ad}_\frac{\partial C}{\partial q} \xi \right) ^\flat {\text{ d }}t \,+ \sum _i \mathrm {ad}^*_{\sigma _i}q \circ dW_t^i = 0\,. \end{aligned} \end{aligned}$$

Recall for the deterministic equations that the energy decays for $\theta >0$ as

3.4 $$\begin{aligned} \frac{\text{ d }}{{\text{ d }}t}h(\mu ,q) = - \,\theta \left\| \mathrm {ad}_\frac{\partial C}{\partial \mu }\xi \right\| ^2 - \theta \, \left\| \mathrm {ad}_\frac{\partial C}{\partial \mu } r+ \mathrm {ad}_\frac{\partial C}{\partial q} \xi \right\| ^2\, , \end{aligned}$$

where the second term is present only in the semidirect product setting (Gay-Balmaz and Holm 2014).

#### Remark 3.1

(Choice of Casimir) The selective decay approach preserves the entire coadjoint orbit, and the speed of decay depends upon which invariant function *C* one uses in implementing it. Indeed, either the first or second term of (3.4) can vanish depending on the choice of Casimir. We refer to the heavy top example in Sect. 6 for more details.

#### Remark 3.2

(Variational principle and reduction) The reader may have noticed already that although we introduced noise via variational principles, the dissipation is added as an extra term in the equations of motions. Indeed, the introduction of noise can also be seen as replacing the Hamiltonian *h* with the stochastic quantity $h\,{\text{ d }}t + \Phi _i \circ dW_t^i$, and we expect to replace it in both the advection and dissipation. In fact, such an equation also fits a variational principle, the so-called Hamilton-Pontryagin principle, combined with Lagrange-d’Alembert for the dissipative force.

#### Proposition 3.3

The variational principle

3.5 $$\begin{aligned} \begin{aligned}&\delta \left( \int l(\xi )\, {\text{ d }}t\right) + \int \left\langle \theta \left[ \frac{\partial C}{\partial \mu }, g^{-1} dg \right] , \left[ \frac{\partial C}{\partial \mu }, g^{-1} \delta g\right] \right\rangle \\&\quad +\, \delta \left( \sum _i \int \langle \mu , g^{-1} dg - \xi {\text{ d }}t - \sigma _i\circ dW_t^i \rangle \right) = 0\, , \end{aligned} \end{aligned}$$

yields the stochastic process

3.6 $$\begin{aligned} \begin{aligned}&d\mu + \mathrm {ad}^*_\frac{\delta h}{\delta \mu } \mu \, {\text{ d }}t + \theta \, \mathrm {ad}^*_\frac{\delta C}{\delta \mu } \left[ \frac{\delta C}{\delta \mu }, \frac{\delta h}{\delta \xi } \right] ^\flat {\text{ d }}t + \theta \,\sum _i \mathrm {ad}^*_\frac{\delta C}{\delta \mu } \left[ \frac{\delta C}{\delta \mu }, \sigma _i \right] ^\flat \circ dW_t^i \\&\quad +\, \sum _i\mathrm {ad}^*_{\sigma _i} \mu \circ dW_t^i = 0 \,, \end{aligned} \end{aligned}$$

for free variations $\delta g, \delta \xi$ and $\delta \mu$.

#### Proof

By a direct computation, using the theory of Hamilton-Pontryagin for the noise and Lagrange-d’Alembert for the double bracket dissipation. $\square$

This term would be admissible since it preserves the coadjoint orbit. However, we do not include it here. Instead, we leave it for treatment elsewhere, as it complicates the calculations to follow without significantly affecting the solution behaviour; since it is proportional to $\theta \sigma ^2$, and $\theta$ and $\sigma ^2$ are taken as being small compared to the original dynamics so that the noise and dissipation are viewed as perturbations.

Interestingly, the Hamilton-Pontryagin principle produces a more physically consistent dissipative equation. This suggests that this principle is the more general than the Clebsch derivation. On top of this remark, we already mentioned that for semidirect product structures, the Clebsch derivation does not allow the noise fields to depend on the advected quantities, whereas the Hamilton-Pontryagin does not have this restriction.

Asymptotically in time, $t\rightarrow \infty$, the deterministic equations with selective decay will tend towards a state which is compatible with the state of minimal energy, as shown in Gay-Balmaz and Holm (2014). However, the presence of noise will balance the dissipation due to selective decay and prevent the system from reaching this deterministic equilibrium position. This feature will imply a non-constant stationary distribution of the corresponding Fokker–Planck solution to be studied in the next section, as well as the existence of random attractors, for which we refer to Kondrashov et al. (2015) and Schenk-Hoppé (1998) for background information.

### The Fokker–Planck Equation and Stationary Distributions

To study the balance between multiplicative noise and nonlinear dissipation, we compute the Fokker–Planck equation associated with the process (3.1) or, equivalently, (3.3), and its stationary solutions. We obtain the Fokker–Planck equation

3.7 $$\begin{aligned} \frac{\text{ d }}{{\text{ d }}t} \mathbb {P}(\mu ) + \{h,\mathbb {P}\} +\theta \left\langle \left[ \frac{\partial \mathbb {P}}{\partial \mu }, \frac{\partial C}{\partial \mu }\right] , \left[ \frac{\partial h}{\partial \mu }, \frac{\partial C}{\partial \mu }\right] \right\rangle - \frac{1}{2} \sum _i \{\Phi _i,\{\Phi _i,\mathbb {P}\}\}=0.\nonumber \\ \end{aligned}$$

The stationary distribution of this Fokker–Planck equation is no longer a constant on the coadjoint orbits. Instead, it now depends on the energy, as summarised in the following theorem.

#### Theorem 3.4

For a compact semi-simple Lie algebra ${\mathfrak {g}}$, let the noise be isotropic, that is $\sigma _i= \sigma e_i$ for an arbitrary $\sigma \in \mathbb {R}$, where the $e_i$’s span the underlying vector space of the dual Lie algebra ${\mathfrak {g}}^*\cong {\mathfrak {g}}$. The stationary distribution of the Fokker–Planck equation (3.7) associated to (3.1) with Casimir $C= \kappa (\mu ,\mu )$ is given by

3.8 $$\begin{aligned} \mathbb {P}_\infty (\mu ) = Z^{-1} e^{-\frac{2\theta }{\sigma ^2} h(\mu )}\, , \end{aligned}$$

where *Z* is the normalisation constant, or partition function, that enforces $\int \mathbb {P}_\infty (\mu ) d\mu = 1$.

#### Proof

The stationary distribution is given by solving $\frac{\text{ d }}{{\text{ d }}t} \mathbb {P}_\infty (\mu )= 0$. The isotropic noise enforces the advection term to vanish independently of the other terms. We therefore use the Ansatz $\mathbb {P}_\infty (\mu ) = f(h(\mu ))$, where the function *f* is to be determined. Consequently, only the selective decay and the double bracket term remain. The selective decay is first rewritten, using the bi-invariance property of the Killing form (2.4), as

$$\begin{aligned} \theta \left\langle \left[ \frac{\partial \mathbb {P}}{\partial \mu }, \frac{\partial C}{\partial \mu }\right] , \left[ \frac{\partial h}{\partial \mu }, \frac{\partial C}{\partial \mu }\right] \right\rangle&= \theta \left\langle \frac{\partial \mathbb {P}}{\partial \mu }, \mathrm {ad}_\frac{\partial C}{\partial \mu } \left[ \frac{\partial C}{\partial \mu }, \frac{\partial h}{\partial \mu }\right] \right\rangle \\&= \theta \, \mathbf d\left( f(h) \mathrm {ad}_\frac{\partial C}{\partial \mu } \left[ \frac{\partial C}{\partial \mu }, \frac{\partial h}{\partial \mu }\right] \right) \, , \end{aligned}$$

where we have used the property that the coadjoint action for semi-simple Lie algebras is divergence-free. (Notice that the exterior derivative $\mathbf d$ is a divergence operation here.) Since $\kappa (\mu ,\mu )$ is a Casimir and $\mu ^\sharp =\frac{\partial C}{\partial \mu }$, we can rewrite the double bracket due to the noise as

$$\begin{aligned} -\frac{1}{2} \sum _i \{\Phi _i,\{\Phi _i,\mathbb {P}\}\}&= -\sigma ^2\frac{1}{2} \sum _i\left\langle \mathrm {ad}^*_{e_i}\frac{\partial C}{\partial \mu }^\flat , \frac{\partial }{\partial \mu } \left\langle \mathrm {ad}^*_{e_i}\frac{\partial C}{\partial \mu }^\flat , \frac{\partial \mathbb {P}}{\partial \mu } \right\rangle \right\rangle \\ \hbox {(From bi-invariance of }\kappa )\quad&= \sigma ^2\frac{1}{2} \sum _i \mathbf d \left( f'(h)\mathrm {ad}_{e_i}\frac{\partial C}{\partial \mu } \left\langle \mathrm {ad}_\frac{\partial h}{\partial \mu }\frac{\partial C}{\partial \mu }, e_i \right\rangle \right) \\&= \sigma ^2\frac{1}{2} \mathbf d \left( f'(h)\mathrm {ad}_{\mathrm {ad}_\frac{\partial h}{\partial \mu }\frac{\partial C}{\partial \mu }}\frac{\partial C}{\partial \mu } \right) \, . \end{aligned}$$

We have used the bi-invariance of the pairing to enforce the relation $\mathrm {ad}^\dagger _\xi \eta :=\mathrm {ad}^*_\xi \eta ^\flat = - \mathrm {ad}_\xi \eta$. See for example (Varadarajan 1984) for more details. The result (3.8) for the equilibrium distribution then follows by comparing the selective decay term with the double bracket term and noticing that the two terms will cancel, provided $f(x) = e^{-{2\theta \,x}/{\sigma ^2}}$. $\square$

The Fokker–Planck equation with dissipation in the semidirect product setting directly gives

3.9 $$\begin{aligned} \begin{aligned}&\frac{\text{ d }}{{\text{ d }}t} \mathbb {P}(\mu ,q) + \{h,\mathbb {P}\} - \frac{1}{2} \sum _i \{\Phi _i,\{\Phi _i,\mathbb {P}\}\}\, \\&\quad +\,\theta \, \left\langle \left[ \left( \frac{\partial \mathbb {P}}{\partial \mu }, \frac{\partial \mathbb {P}}{\partial q}\right) , \left( \frac{\partial C}{\partial \mu }, \frac{\partial C}{\partial q}\right) \right] , \left[ \left( \frac{\partial h}{\partial \mu },\frac{\partial h}{\partial q}\right) ,\left( \frac{\partial C}{\partial \mu },\frac{\partial C}{\partial q}\right) \right] \right\rangle =0 \,. \end{aligned} \end{aligned}$$

Consequently, for semidirect products, we have the analogue of the previous theorem, but for the marginal stationary distribution on the advected quantities.

#### Theorem 3.5

Provided that the Hamiltonian is of the form $h(\mu ,q)= K(\mu )+V(q)$ for two functions *K* and *V* and that the noise is isotropic, the stationary marginal distribution $\mathbb {P}_\infty ^2(q)$ with the selective decay from the Casimir $\kappa (\mu ,q)$ is given by

3.10 $$\begin{aligned} \mathbb {P}_\infty ^2(q)= Z^{-1} e^{-\frac{2\theta }{\sigma ^2} V(q)}\, , \end{aligned}$$

where *Z* is the normalisation constant, or partition function.

#### Proof

The proof here is similar to the proof for Theorem 3.4. Thus, we only show the main calculations. First, the selective decay term is given explicitly, using the Casimir $\kappa (\mu ,q)$, by

$$\begin{aligned} \theta \, \left\langle \mathrm {ad}_\frac{\partial \mathbb {P}}{\partial \mu } q , \mathrm {ad}_\xi q \right\rangle + \theta \, \left\langle \mathrm {ad}_\frac{\partial \mathbb {P}}{\partial \mu }\mu + \mathrm {ad}_\frac{\partial \mathbb {P}}{\partial q}q , \mathrm {ad}_\xi \mu + \mathrm {ad}_rq \right\rangle \, . \end{aligned}$$

Integrating the selective decay term of (3.9) in $\mu$ and assuming $\mathbb {P}^2(q)= f(V(q))$, gives

$$\begin{aligned} \theta \, \left\langle \mathrm {ad}_\frac{\partial \mathbb {P}^2}{\partial q} q , \mathrm {ad}_r q \right\rangle&= - \theta \left\langle \mathrm {ad}^*_{\mathrm {ad}_r q} q, \frac{\partial \mathbb {P}^2}{\partial q} \right\rangle = - \theta \mathbf d \left( \mathrm {ad}^*_{\mathrm {ad}_r q} q f \right) \, , \end{aligned}$$

where we have used the bi-invariance property of $\kappa$ (2.4), as well as the divergence-free property of the coadjoint action. Then, after integration over $\mu$, the double bracket term becomes

$$\begin{aligned} -\frac{1}{2} \sigma ^2 \mathbf d \left( \mathrm {ad}^*_{e_i} q \left\langle \mathrm {ad}^*_{e_i}q ,\frac{\partial \mathbb {P}^2}{\partial q}\right\rangle \right)&= \frac{1}{2} \sigma ^2 \mathbf d \left( f' \mathrm {ad}^*_{e_i}q \left\langle \mathrm {ad}_q r, e_i \right\rangle \right) \\&= - \frac{1}{2} \sigma ^2 \mathbf d \left( f' \mathrm {ad}^*_{\mathrm {ad}_rq }q \right) \, , \end{aligned}$$

upon again using bi-invariance. Thus, the result follows, as *f* must satisfy $\theta f =\frac{1}{2} \sigma ^2 f'$. $\square$

In the Euler–Poincaré setting, the stationary distribution was concentrated around the positions of minimum energy, and here the advected quantity *q* is concentrated around the position of minimal potential energy. We conjecture that the complete stationary distribution is concentrated around the minimal energy region, as in the Euler–Poincaré setting. However, we will not investigate this conjecture here, as we will be mainly interested in the dynamics of the advected quantities.

#### Remark 3.6

(Gibbs measure) This calculation only uses the bi-invariance of the Killing form, which holds in general for semi-simple Lie algebras. Therefore, the same conclusion applies for other Lie algebras which admit a bi-invariant pairing. In statistical physics, the invariant measure (3.8) is often called a Gibbs measure. This association provides a natural identification of the quantity $\sigma ^2/ 2k_B\theta$ with a Kelvin temperature *T*, where $k_B$ is the Boltzmann constant. This notion of temperature arises via coupling the system with a heat bath, at temperature *T*. Such an open system in statistical physics is referred to as a canonical ensemble, whereas the system without dissipation is closed, and hence fits in the traditional category of micro-canonical ensembles.

#### Remark 3.7

(Non-isotropic noise) To obtain the Gibbs measure, we had to assume an isotropic noise, but stationary distribution also exists with non-isotropic noise. The exact form of these distribution is more involved but will remain close to be a Gibbs measure. We will not compute it here, and only show an example of such distribution in the case of the rigid body, in Sect. 5.

#### Remark 3.8

(Time reversal) The results of this section also hold when evolving backwards in time, using the change of variable $t\rightarrow -t$. Indeed, since the noise *dW* is centred, only the dissipation will be affected by time reversal, and it will have the opposite effect, namely the system will tend towards the highest energy equilibrium position.

## Random Attractors

We now turn to the study of the existence of random attractors (RAs) in our stochastic dissipative systems, in connection with the theory of random dynamical systems (RDS). The classic approach in studying the effect of stochastic forcing of nonlinear dynamical systems proceeds by integrating the system forward in time and performing averages, then studying the Fokker–Planck equation, as we have done up to now. Another approach studies random dynamical systems via the so-called pull-back method. We will not fully explore the theory of random dynamical systems and pullback attractors here. Instead, we will only invoke the main results from the theory and refer the interested reader to Crauel and Flandoli (1994), Crauel et al. (1997), Arnold (1995), Bonatti et al. (2006) and Kloeden and Rasmussen (2011) for in-depth accounts of these subjects. In a nutshell, for a given fixed realisation of the noise, the average is taken over the initial conditions. The noise makes the system time-dependent; so the notion of an attractor should be defined in the pull-back sense, such that for large times the attractive set does not depend on time. That is, the pullback attractor is defined by pulling back a given set of initial conditions from $t=0$ to $t\rightarrow -\infty$ and letting the system evolve to $t=0$. In this limit, the set obtained at $t=0$ is the pullback attractor. In random dynamical systems theory, the pullback attractor is usually called a random attractor, and if it is not singular, it may admit a particular type of measure, the Sinai-Ruelle-Bowen measure (SRB), which is also called a physical measure, see Young (2002). We will denote the physical measure by $\mathbb {P}_\omega (\mu )$ for a given realisation of the noise $\omega$. There is a fundamental relation between this SRB measure and the stationary distribution of the Fokker–Planck equation which was first discovered in Crauel (1991) and later in a theorem of Crauel and Flandoli (1998). This relation is informally given by

4.1 $$\begin{aligned} \int _\Omega \mathbb {P}_\omega (\mu ) d\omega = \mathbb {P}_{\infty }(\mu )\, , \end{aligned}$$

for the probability space $\Omega$. Here we are referring to probability densities, and the SRB measure can be seen as the invariant measure most compatible with volume, although volume in phase space is not preserved, because of dissipation. For more explanation, see Young (2002).

### Remark 4.1

(Periodic kicking) We are only considering here the interaction of noise and dissipation. However, if the noise were replaced by a simpler deterministic forcing, similar results would emerge. In particular, periodic forcing or kicking of dissipative dynamical systems has been studied in great detail in numerous works, e.g., in Lin and Young (2010) and Lu et al. (2013). In Sect. 5.5, we will implement periodic kicking and damping in the rigid body, and will numerically demonstrate the existence of non-singular attractors and chaos. We have left deeper theoretical studies of these systems for future investigations.

### Existence of Attractors

We first determine that the stochastic processes (3.1) and (3.3) do indeed admit random attractors, provided the top Lyapunov exponent is positive. See Kondrashov et al. (2015) and Schenk-Hoppé (1998) and references therein for more details about this type of approach. Then, we will estimate the value of the top Lyapunov exponent using numerical simulations for the rigid body in Sect. 5.

#### Theorem 4.2

The stochastic process (3.1) admits a random attractor, for every Lie group *G*.

#### Proof

The SDE (3.1) may be recast as a random dynamical equation (RDE) by using the following vector Wiener processes $z_i$,

4.2 $$\begin{aligned} dz_i = \sigma _i dW_t^i\,, \end{aligned}$$

where $z_i$ is understood as a vector process in the direction along $\sigma _i$. In the sequel, we will denote $z(t,\omega )= \sum _i z_i(t,\omega )\in {\mathfrak {g}}$. The process *z*(*t*) thus defines a random path in the Lie algebra ${\mathfrak {g}}$ and, via the exponential map, a random path in the group *G* as $g(t,\omega )= e^{z(t,\omega )}$.

We then define a new variable $\widetilde{\mu }(t)= g(t)\mu (t) := \mathrm {Ad}^*_{g(t)}\mu (t)$ and we have, from (3.1) (see for example Marsden and Ratiu 1999),

$$\begin{aligned} d\widetilde{\mu }(t)&= \mathrm {Ad}^*_{g(t)} \left( -\sum _i\mathrm {ad}^*_{\sigma _i}\mu \circ dW + d\mu \right) = \mathrm {Ad}^*_{g(t)} \left( F(\mathrm {Ad}^*_{g(t)^{-1}}\widetilde{\mu }(t))\right) {\text{ d }}t\, , \end{aligned}$$

where our stochastic process is generically written $d\mu = F(\mu ){\text{ d }}t + G_i(\mu )\circ dW_i$ for convenience. From here, we have the RDE associated to (3.1) of the form

4.3 $$\begin{aligned} \frac{\text{ d }}{{\text{ d }}t}\widetilde{\mu }(t) = \widetilde{F}(\widetilde{\mu }(t),g(t))\, , \end{aligned}$$

where $\widetilde{F}$ is defined in the previous calculation as the drift part of the process. Recall that from the theory of selective decay we have (Gay-Balmaz and Holm 2013)

$$\begin{aligned} \frac{\text{ d }}{{\text{ d }}t}h(\mu ) = -\,\theta \left\| \left[ \frac{\partial C}{\partial \mu },\frac{\partial h}{\partial \mu }\right] \right\| ^2\, , \end{aligned}$$

and $h(\mathrm {Ad}^*_g\mu ) = h(\mu )$, because *h* is *G*-invariant, so that this equality becomes for (4.3),

4.4 $$\begin{aligned} \frac{\text{ d }}{{\text{ d }}t}h(\widetilde{\mu }) = -\theta \left\| \left[ \mathrm {Ad}_{g^-1}\frac{\partial C(\widetilde{\mu })}{\partial \widetilde{\mu }}, \mathrm {Ad}_{g^-1}\frac{\partial h(\widetilde{\mu })}{\partial \widetilde{\mu }}\right] \right\| ^2 \le 0\,. \end{aligned}$$

This inequality assures that the energy decays at a random strictly negative rate. The existence of the random attractor then follows from standard arguments, demonstrated, for example, in the linear case by Schenk-Hoppé (1998) and in a more general nonlinear setting by Chekroun et al. (2011). $\square$

The idea of this proof is to generalise the linear change of variables used to recast the original stochastic process as a random dynamical equation, by using a nonlinear group theoretical change of variable. The dissipative property follows from the selective decay theory and the invariance of the Hamiltonian under the group action. This theorem is general, in that no specific assumptions on the Lie group need to be imposed. In particular, modulo difficulties in analysis, the theorem should also apply for the diffeomorphism group used in the description of compressible fluid equations. However, we have no intention of investigating the infinite dimensional theory here.

The same result persists in the semidirect product theory, as developed earlier.

#### Corollary 4.2.1

Theorem 4.2 applies to semidirect product stochastic processes (3.3).

#### Proof

The proof follows the same argument, upon using the action of the group *G* and the Lie algebra ${\mathfrak {g}}$ and the advected quantities in *V* to define the change of variables. The decay rate of the energy is given by using the deterministic selective decay formulae (3.4). $\square$

### Existence of the SRB Measure

We now turn to the existence of the SRB measure. Theorem 4.3 below for the existence of SRB measures will invoke Hörmander’s theorem about the smoothness of transition probabilities for a diffusion satisfying the so-called Hörmander (Lie) bracket conditions. The Lie bracket [*v*, *w*](*x*) of two vector fields *v*(*x*), *w*(*x*) in $\mathbb {R}^n$ is defined as

4.5 $$\begin{aligned} {[}v,w](x) = Dv(x)w(x) - Dw(x)v(x)\, , \end{aligned}$$

where we denote by *Dv* the derivative matrix given by $(Dv)_{ij} = \partial _j v_i = v_{i,j}$. Given an SDE of the form

4.6 $$\begin{aligned} dx = A_0(x){\text{ d }}t + \sum A_i(x)\circ dW_t^i\, , \end{aligned}$$

the Hörmander condition we use states that if the following condition is satisfied

4.7 $$\begin{aligned} \cup _{k\ge 1}\, V_k(x) = \mathbb {R}^n,\quad \hbox {for all}\quad x\,, \end{aligned}$$

where

4.8 $$\begin{aligned} \begin{aligned} V_k(x)&= V_{k-1}(x) \cup \text{ span }\{ [v(x),A_j(x)]: v\in V_{k-1},j\ge 0\}\quad \mathrm {and}\\ V_0(x)&= \text{ span }\{ A_j, j \ge 1 \} \,, \end{aligned} \end{aligned}$$

then the invariant measure of (4.6) is smooth with respect to the Lebesgue measure. The Hörmander condition implies the following standard theorem for stochastic dissipative systems.

#### Theorem 4.3

If the largest Lyapunov exponent of (3.1) is positive, the random attractor is the support of a Sinai-Ruelle-Bowen (SRB) measure.

#### Proof

The proof uses the corollary of Theorem B in (Ledrappier and Young 1988), which assumes the existence of a random attractor. The only point left to show here is that the parabolic Hörmander condition (4.7) is fulfilled. Given the Stratonovich process (4.6) in $\mathfrak {g}^*$, we only need to check that the vector fields $A_1,\dots ,A_N$ will span the tangent space to the coadjoint orbits as long as *N* is sufficiently large. Since $A_i(\mu ):= \text{ ad }^*_{\sigma _i} \mu$, they are tangent to the coadjoint orbits. The minimal number of $A_i$ needed cannot be found, in general, as it will depend on the Lie symmetry algebra and the form of the Hamiltonian. Nevertheless, the $\sigma _i$ span the vector space ${{\mathfrak {g}}}$, and the Hörmander condition is fulfilled. $\square$

#### Corollary 4.3.1

Theorem 4.3 also applies for the semidirect product case, even with $\eta _i= 0$.

#### Proof

The same argument applies here, even if $\eta _i=0$, as the semidirect product structure will automatically span the whole space, provided ${\mathfrak {g}}$ is already spanned, and *h* is not too degenerate on *V*. $\square$

### Estimation of Lyapunov Exponents

In principle, it is possible to compute the value of the top Lyapunov exponent as a function of the parameters of the system. However, this turns out to be a very challenging computation. Nonetheless, positivity of the top Lyapunov exponent is important to determine, as it allows us to use the previous Theorem 4.3 to prove the existence of a non-singular random attractor with an SRB measure and positive entropy. We will restrict ourselves to the first step of the calculation and explain why it is a difficult problem. We will then estimate the top Lyapunov exponent numerically in the example section, and leave the rigorous proof as an open problem.

The very first step is to estimate the sum of the Lyapunov exponents using the multiplicative ergodic theorem (MET), that we state here in its simplest form. We will denote by $\Omega$ the probability space of the stochastic dynamical system.

#### Theorem 4.4

(MET theorem) For an ergodic stochastic process $\mu$ with invariant measure $\mathbb {P}_\infty$ there exists an flow invariant subset $\Delta \subset {\mathfrak {g}}^*\times \Omega$ of the *n*-dimensional phase space and a sequence of ordered Lyapunov exponents $\lambda _i$ for $i= 1, \ldots , n$ such that the following properties hold for all $(\mu , \omega ) \in \Delta$:

1. The dual of the Lie algebra can be decomposed into a direct sum
  $$\begin{aligned} T_\mu {\mathfrak {g}}^*= E_1(\omega , \mu ) \oplus \ldots \oplus E_n(\omega , x),, \end{aligned}$$
  where
  4.9 $$\begin{aligned} \delta \mu \in E_i \Leftrightarrow \lim _{t\rightarrow \infty } \frac{1}{t}\mathrm {log}\Vert DF(t,\omega , \mu ) \delta \mu \Vert = \lambda _i\, . \end{aligned}$$
  $d(\delta \mu ) = DF(t,\omega , \mu ) \delta \mu$ is the linearisation of the flow equation $d\mu = F(t,\omega , \mu )$ along a particular solution $\mu (t)$.
2. For a generic $\delta \mu$, the associated Lyapunov exponent is $\lambda (\omega , x, v) = \lambda _+$, the largest one almost surely.
3. The sum of the Lyapunov exponents is
  4.10 $$\begin{aligned} \sum _i \lambda _i= \lim _{t\rightarrow \infty } \frac{1}{t} \mathrm {log}\, \mathrm {det}\, DF (t, \omega , \mu )\, . \end{aligned}$$

For simplicity here,, we have assumed that the multiplicity of the Lyapunov exponents is always 1; that is, they are all distinct. We refer to Arnold (1995) for more details on this theorem and its generalisations. The stochastic systems which we consider here are written on compact semi-simple Lie algebras, such that $c^2=\Vert \mu \Vert ^2$ is constant and defines a bounded set. The energy functional $h(\mu )$ is also a generic quadratic kinetic energy term, with a given inertia tensor $\mathbb {I}^{-1}$, corresponding to the Hessian matrix of $h(\mu )$. We can then prove the following formula for the sum of the Lyapunov exponents.

#### Proposition 4.5

Provided the Lie algebra is compact semisimple, the sum of the Lyapunov exponents is estimated from below by

4.11 $$\begin{aligned} \sum _i d_i\lambda _i \ge - \frac{1}{2} |\epsilon | n\sigma ^2 -\theta |\epsilon |\left( c^2 \mathbb {I}_\mathrm {min}^{-1}- \mathbb {E}_\infty h(\mu )\right) \, , \end{aligned}$$

where $c= \Vert \mu \Vert ^2$, $\epsilon$ is the Killing form constant, *n* is the number of $\sigma _i=\sigma e_i$ spanning the Lie algebra and $d_i$ are the multiplicity of each Lyapunov exponent. Thus, the dimension of the Lie algebra, $n=\mathrm{dim}(\mathfrak {g})$. The quantity $\mathbb {I}_\mathrm {min}^{-1}$ is the largest eigenvalue of the Hessian of the Hamiltonian. The expectation $\mathbb {E}_\infty$ is taken with respect to the invariant measure $\mathbb {P}_\infty$. An estimation from above is also available, upon using $\mathbb {I}_\mathrm {max}^{-1}$, the minimal eigenvalue, instead of $\mathbb {I}_\mathrm {min}^{-1}$.

#### Proof

Let us denote the stochastic process (3.1) in Itô form by

$$\begin{aligned} d\mu = F(\mu ) {\text{ d }}t + \sum _iG_i(\mu )dW_t^i\, . \end{aligned}$$

We can now directly apply the MET Theorem 4.4 to compute the sum of the Lyapunov exponents

4.12 $$\begin{aligned} \sum _i\lambda _i = \lim _{t\rightarrow \infty } \frac{1}{t} \mathrm {log}\, \mathrm {det}\, \delta \mu (t,\omega ,x)\, . \end{aligned}$$

We can then use Jacobi formula to rewrite (4.12) as

4.13 $$\begin{aligned} \lim _{t\rightarrow \infty } \frac{1}{t} \mathrm {log}\, \mathrm {det}\, \delta \mu (t,\omega ,x)= \lim _{t\rightarrow \infty } \frac{1}{t} \mathrm {Tr}\int ^t DF(\varphi (t,x,\omega ))ds\, . \end{aligned}$$

Here, we have intentionally dropped the linearisation of the noise amplitude, since this term will vanish (the trace of the adjoint action always vanishes). Finally, ergodicity of this process gives

4.14 $$\begin{aligned} \sum _i\lambda _i = \int \mathrm {Tr}(DF(\mu )) \mathbb {P}_\infty (\mu ) d\mu \,, \end{aligned}$$

where $\mathbb {P}_\infty$ is the invariant measure of the underlying stochastic process. The calculation of the trace simplifies in the case of a compact semi-simple Lie algebra with the Killing form $\mathrm {Tr}(\mathrm {ad}_A\mathrm {ad}_B)= \epsilon A\cdot B$, where $\epsilon < 0$ depends on the Lie algebra. Then, using the explicit form of *F* along with semi-simplicity for ${\mathfrak {g}}$, yields

$$\begin{aligned} F(\mu ) = \mathrm {ad}_\frac{\partial h}{\partial \mu } \mu + \theta \, \mathrm {ad}_\mu \mathrm {ad}_\mu \frac{\partial h}{\partial \mu } + \frac{1}{2}\sum _i \mathrm {ad}_{\sigma _i}\mathrm {ad}_{\sigma _i}\mu \, . \end{aligned}$$

Consequently, we arrive at

4.15 $$\begin{aligned} \mathrm {Tr}(DF(\mu ))&= \mathrm {Tr}\left( - \theta \, \mathrm {ad}_\mu \mathrm {ad}_\frac{\partial h}{\partial \mu } + \theta \, \mathrm {ad}_\mu \mathrm {ad}_\mu \frac{\partial ^2h }{\partial \mu ^2} + \frac{1}{2}\sum _i \sigma ^2 \mathrm {ad}_{e_i}\mathrm {ad}_{e_i} \right) \end{aligned}$$

4.16 $$\begin{aligned}&= |\epsilon | \theta h(\mu )+\theta A(\mu ,\mu ) - \frac{1}{2}|\epsilon | n \sigma ^2\, , \end{aligned}$$

where *n* is the number of $\sigma _i$ fields. The $A(\mu ,\mu )$ term depends on the Lie algebra structure constants and is difficult to obtain explicitly for every compact semi-simple Lie algebra. However, we can estimate it here using

4.17 $$\begin{aligned} |\epsilon |\theta \kappa (\mu ,\mu ) \mathbb {I}^{-1}_\mathrm {max}\ge \theta \mathrm {Tr}\left( \theta \mathrm {ad}_\mu \mathrm {ad}_\mu \frac{\partial ^2 h}{\partial \mu ^2}\right) \ge |\epsilon |\theta \kappa (\mu ,\mu )\mathbb {I}^{-1}_\mathrm {min}\, . \end{aligned}$$

Collecting terms then gives a lower and upper bound for the sum of the Lyapunov exponents (4.11). $\square$

Unfortunately, Proposition 4.5 only gives a negative lower bound for the sum of the Lyapunov exponents of an arbitrary compact semi-simple Lie algebra. Indeed, the last term can be bounded from above by

$$\begin{aligned} \mathbb {E}_\infty h(\mu ) = \int h(\mu ) e^{-\frac{2\theta }{\sigma ^2}h(\mu )}d\mu \le c^2 \mathbb {I}_{\mathrm {min}}^{-1}\, . \end{aligned}$$

Thus, the sum of the Lyapunov exponents is found to be negative.

A precise value can be computed explicitly for each Lie algebra, by using the structure constants to calculate the term $A(\mu ,\mu )$ in (4.16). We will show this calculation in the case of *SO*(3) in the free rigid body example in Sect. 5.

#### Remark 4.6

(Non-compact Lie algebras) This argument does not apply for non-compact semi-simple Lie algebras, as the Killing form is not sign-definite, so it does not provide us with a norm.

Having only a negative lower bound for the sum of the Lyapunov exponent means we must proceed further by estimating the top Lyapunov exponent, to obtain an SRB measure. Obtaining a precise estimate for our general class of system is still an open problem, but we will show here the main steps towards this result. We will then numerically estimate it for the simple case of *SO*(3) in the Sect. 5.

First, recall the linearisation of the flow, in Itô form

4.18 $$\begin{aligned} d\delta \mu = DF(\mu ) \delta \mu \, {\text{ d }}t + \sum _i DG_i(\mu ) \delta \mu \,dW_t^i\, , \end{aligned}$$

where $DG_i(\mu ) = \mathrm {ad}_{\sigma _i}$. We then want to apply the Furstenberg-Kasminskii formula. (See Arnold 1995 for details.) For this purpose, we introduce the following change of variables

4.19 $$\begin{aligned} R= \Vert \delta \mu \Vert \in \mathbb {R}\quad \mathrm {and}\quad \Phi = \frac{\delta \mu }{\Vert \delta \mu \Vert } \in \mathbb {S}^{n-1}\, , \end{aligned}$$

where $\mathbb {S}^{n-1}$ is the (*n*-1)-sphere. The corresponding stochastic processes are

4.20 $$\begin{aligned} d R&= \langle \Phi , (DF(\mu ){\text{ d }}t + DG_idW^i_t)\Phi \rangle R := Q(\mu ,\Phi ) R\,, \end{aligned}$$

4.21 $$\begin{aligned} d \Phi&= (DF(\mu ){\text{ d }}t + DG_idW^i) \Phi - \langle \Phi , (DF(\mu ){\text{ d }}t + DG_idW^i ) \Phi \rangle \Phi \nonumber \\&:= P(\mu ,\Phi ){\text{ d }}t + \sum _i P_i(\mu ,\Phi )dW_t^i\, . \end{aligned}$$

As a direct consequence of ergodicity and the multiplicative ergodic Theorem 4.4 [Item 1 and 2], the Furstenberg-Kasminskii formula then gives the top Lyapunov exponent by evaluating the following integral

4.22 $$\begin{aligned} \lambda _+ = \int Q(\mu ,\Phi ) \mathbb {Q}(\mu ,\Phi )d\mu d \Phi \, , \end{aligned}$$

where $\mathbb {Q}(\mu ,\Phi )$ is the joint stationary distribution of the processes for $\mu$ and $\Phi$, where the processes for $\Phi$ depend on $\mu$, but not the reverse.

One can see that a rough estimate of the integrand from below would always be negative. Consequently, we need more work to obtain a positive bound. Let us evaluate the quantity *Q* at the positions $(\mu _i,\Phi _j)$, where we use the basis corresponding to the eigenvalues of the Hessian of *h*, i.e., the inverse of the moment of inertia $\mathbb {I}^{-1}$. We also order the eigenvalues as $\mathbb {I}_1>\ldots >\mathbb {I}_n$. Note that because the dynamics of $\mu$ takes place on the coadjoint orbit, the linearisation is tangent to it and thus $i\ne j$ for the choice of positions $(\mu _i,\Phi _j)$. A direct calculation gives the following simplifications of the quantity $Q(\mu _i,\Phi _j)$

$$\begin{aligned} Q(\mu _i,\Phi _j)&= \langle \Phi _j, DF(\mu _i)\Phi _j\rangle \\&= \langle \mathrm {ad}_{\mu _i}\Phi _j, \mathbb {I}^{-1} \Phi _j\rangle -\frac{1}{2}\sigma ^2\sum _k \langle \mathrm {ad}_{e_k}\Phi _j,\mathrm {ad}_{e_k}\Phi _j\rangle \\&\quad +\, \theta \, \langle \mathrm {ad}_{\mu _i}\Phi _j,\mathrm {ad}_{\mathbb {I}^{-1}\mu _i}\Phi _j\rangle - \theta \, \langle \mathrm {ad}_{\mu _i}\Phi _j,\mathrm {ad}_{\mu _i}\mathbb {I}^{-1} \Phi _j\rangle \\&= \langle \mu _i,\mathrm {ad}_{\Phi _j} \mathbb {I}^{-1} \Phi _j\rangle -\frac{1}{2}\sigma ^2\sum _k \Vert \mathrm {ad}_{e_k}\Phi _j\Vert ^2 \\&\quad +\, \theta \, \langle \mathrm {ad}_{\mu _i}\Phi _j,\mathrm {ad}_{\mu _i}(\mathbb {I}^{-1}_i\mathrm {Id}-\mathbb {I}^{-1}) \Phi _j\rangle \\&= -\frac{1}{2}\sigma ^2\sum _k \Vert \mathrm {ad}_{e_k}\Phi _j\Vert ^2+ \theta \, (\mathbb {I}^{-1}_i -\mathbb {I}^{-1}_j)\Vert \mathrm {ad}_{\mu _i}\Phi _j\Vert ^2\\&= -\frac{n-1}{2}\sigma ^2 + \theta \, c^2(\mathbb {I}^{-1}_i -\mathbb {I}^{-1}_j)\, , \end{aligned}$$

where we have used the Casimir sphere radius *c* (thus $\mu _i = c\, e_i$) and the fact that $\Vert \Phi \Vert ^2= 1$. The last formula conveys a lot of information about the possible sign for $\lambda _+$. Indeed, depending on the choice of *i* and *j*, it is possible that $Q(\mu _i,\Phi _j)$ is positive, provided the difference between the moments of inertia is large enough with respect to the $\sigma$, $\theta$ and *c*. That difference between the moments of inertia is important and is tied to the nature of the random attractor. Indeed, differences in the moments of inertia imply different speeds for nearby orbits, and thus a shear effect. This shear effect is a common source of random attractors, which has appeared in a number of recent papers such as (Wang and Young 2003; Lin and Young 2008). The necessity of sufficiently large shear for the existence of the random attractor is thus implied by the last formula in the computation above. Of course, this is not the whole story, as the original dynamics of the system is also an important factor. Indeed, without noise and dissipation, the system is Hamiltonian. Thus the sum of Lyapunov exponents vanishes in this case, and the top Lyapunov exponent must be positive for a large set of initial conditions. In the present case, integrating the deterministic part of *Q* against the Gibbs measure is already analytically difficult, and no particular sign can be easily expected from examining this term. The only thing we can expect at this stage is that for large dissipation when the Gibbs measure is localised around the equilibrium positions of minimum energy, the dynamics of the deterministic system is negligible, whereas for a small dissipation the deterministic dynamics will be important. We refer to Sect. 5, where we will evaluate the top Lyapunov exponent in the rigid body example by numerical simulation.

We now turn to the semidirect product structure and also estimate the sum of the Lyapunov exponents in the following proposition, where we choose to use the Casimir $C(\mu ,q)= \frac{1}{\epsilon }\kappa (q,q)=c^2$ for the dissipative term for simplicity only.

#### Proposition 4.7

The sum of the Lyapunov exponents for the semidirect stochastic process (3.3) with Casimir $C(\mu ,q)= \frac{1}{\epsilon }\kappa (q,q)=c^2$ is given by

4.23 $$\begin{aligned} \sum _i \lambda _i \ge - |\epsilon |\, n\sigma ^2 -\theta c^2 \mathbb {I}_\mathrm {min}^{-1}\, . \end{aligned}$$

#### Proof

We follow the proof for the Euler–Poincaré case. Let us denote the stochastic process (3.3) in Itô form by

$$\begin{aligned} d(\mu ,q) = \left[ F^\mu (\mu ,q) +F^q(\mu ,q)\right] {\text{ d }}t + \sum _i\left[ G_i^\mu (\mu ,q)+G_i^q(\mu ,q)\right] dW_t^i\, , \end{aligned}$$

where we denoted $F^\mu$ (resp. $F^q$) the $\mu$ (resp. *q*) component of *F*. The MET theorem, Jacobi’s formula and ergodicity of this process gives

4.24 $$\begin{aligned} \sum _i\lambda _i = \int \mathrm {Tr}\left( D_\mu F^\mu (\mu ,q) + D_qF^q(\mu ,q)\right) \mathbb {P}_\infty (\mu ,q) d(\mu ,q)\, , \end{aligned}$$

where $\mathbb {P}_\infty (\mu ,q)$ is the invariant measure of the underlying stochastic process, and $D_\mu$ and $D_q$ denote the Jacobian matrices taken with respect to $\mu$ or *q*, respectively. Consequently, after substituting the Casimir *C*(*q*) in the general formula (3.3), we obtain

$$\begin{aligned} \mathrm {Tr}(D_\mu F^\mu +D_qF^q) = \mathrm {Tr}\left( -\theta \, \mathrm {ad}_q\mathrm {ad}_q\mathbb {I}^{-1} -\sigma ^2 \sum _i\mathrm {ad}_{e_i}\mathrm {ad}_{e_i} \right) \, , \end{aligned}$$

and, using again (4.17), we have the result (4.23). $\square$

As before, the sum of the Lyapunov exponents is negative. Thus, we must estimate the top Lyapunov exponent to prove the existence of a non-singular SRB measure for this system. The same difficulty as before remains in this case for estimating the top Lyapunov exponent using the Furstenberg-Kasminskii formula.

*Summary* In this section, we have studied the interaction of multiplicative noise and nonlinear dissipation on coadjoint orbits. For this purpose, we added a double bracket dissipation mechanism to the previously derived stochastic process to preserve the coadjoint orbit structure on which the solutions of the stochastic process are supported. In the case of semi-simple Lie algebras, we obtained the invariant measure of the Fokker–Planck equation and found the associated Gibbs measure on the coadjoint orbits. In the semidirect product case, this result was shown to hold for the marginal distribution of the advected quantity only, where the Gibbs distribution depends only on the potential energy. We then proved the existence of random attractors for a wide class of systems by using the dissipative property of the double bracket and the Hörmander condition on the generating vector fields, provided the top Lyapunov exponent is positive. Unfortunately, we were not able to derive an exact lower positive bound for the top Lyapunov exponent. However, numerical investigations in the example Sects. 5 and 6 will provide us with strong pieces of evidence of the positivity of the top Lyapunov exponent for some region of the parameter space $(\theta , \sigma )$. In the next two sections, we will study two specific examples of stochastic deformations of the Euler–Poincaré dynamical equation, for the free rigid body and the heavy top, using both analytical and numerical tools.

## Euler–Poincaré Example: The Stochastic Free Rigid Body

This section introduces stochastic dynamics for the classic example of the Euler–Poincaré dynamical equation; namely, the equation for free rigid body motion. Stochastic rigid body models have arisen in various fields of application, such as nanoparticles (Shrestha et al. 2015; Blum et al. 2006), molecular biology (Gordon et al. 2009), polymer dynamics (Chirikjian 2009[Section 13.7]), filtering in aeronautics: guidance and tracking (Willsky 1974). We refer to Chirikjian (2012) and Chirikjian (2009) for more applications. One source of models for stochastic dynamics stems from the so-called rotational Brownian motion of molecules. Rotational Brownian motion comprises the random change in the orientation of a polar molecule due to collisions with other molecules and is an important element in the theory of dielectric materials. Perrin and Debye’s non-inertial theories are the most well-known models, see for example Chirikjian (2009)[section 16.3]. Rotational Brownian motions have also been observed in a laboratory setting and have been properly documented in Han et al. (2006). Much of the current research in rotational Brownian motions has been devoted to inertial models, non-spherical molecules and the possibility of dipole-dipole interactions. Walter et al. (2010) took a step further in proposing an inertial, Langevin type of generalisation to the rigid body equations aiming at studying systems of rigid bodies as models for polymer dynamics. The coupling between linear and rotational dynamics was important in this case, to capture the motion features of long polymeric chains. Their models assume linearity in the noise for both linear and angular momentum variables, whereas the model used here is fully nonlinear with multiplicative noise and preserves strong geometrical features such as the coadjoint orbits.

### Remark 5.1

(The LLG equation) We mention that the stochastic Landau-Lipschitz-Gilbert (LLG) equation studied for example in Garanin (1997), Brzeźniak et al. (2012) and Kohn et al. (2005) has the same structure as our stochastic dissipative rigid body equation. Indeed, we preserve the coadjoint orbit, thus the amplitude of the momentum variable, which corresponds to the strength of magnetic moments in the LLG model. We will not study this link further here as the LLG equation is a PDE in two or three dimensions and requires different analytical methods than the rigid body equation.

### The Stochastic Rigid Body

The canonical example for illustrating the Euler–Poincaré reduction by symmetry is the free rigid body, whose configuration space is the group of rotations *SO*(3). For a complete treatment from the viewpoint of reduction, we refer to Marsden and Ratiu (1999), For simplicity here, we rely on the isomorphism $\mathfrak { so}(3)\cong \mathbb {R}^3$ which translates the commutator in the Lie algebra to the cross product of three-dimensional vectors, via $[A , B ] \rightarrow \varvec{A}\times \varvec{B}$, where $\mathbb {R}^3$ vectors are denoted with bold font. This map allows us to use a slightly different Killing form than the canonical one. Namely, we shall use the scalar product as our pairing, via the formula $\varvec{A}\cdot \varvec{B}= -\frac{1}{2} \kappa (A,B)$.

The reduced Lagrangian of the free rigid body is written in terms of the angular velocity $\Omega \in \mathfrak {so}(3)$ and a prescribed moment of inertia $\mathbb {I}\in \mathrm {Sym}(3)$ as

5.1 $$\begin{aligned} l(\varvec{\Omega }) = \frac{1}{2} \varvec{\Omega } \cdot \mathbb { I} \varvec{\Omega } : = \frac{1}{2} \varvec{\Omega }\cdot \varvec{\Pi }\,, \end{aligned}$$

where the angular momentum $\varvec{\Pi }$ is defined accordingly and the Legendre transform gives the reduced Hamiltonian $h(\varvec{\Pi }) = \frac{1}{2} \varvec{\Pi } \,\mathbb {I} ^{-1}\varvec{\Pi }$. We take the stochastic potential to be linear in the momentum variable $\varvec{\Pi }$

5.2 $$\begin{aligned} \Phi _i(\varvec{\Pi }) = \sum _{i=0}^3 \varvec{\sigma }_i\cdot \varvec{\Pi }\,, \end{aligned}$$

where the constants $\varvec{\sigma }_i$ generically span $\mathbb {R}^3$ but can be chosen in various ways. The stochastic process for $\varvec{\Pi }$ is then computed from (2.8) to be

5.3 $$\begin{aligned} d\varvec{\Pi } + \varvec{\Pi }\times \varvec{\Omega }\, {\text{ d }}t + \sum _i\varvec{\Pi }\times \varvec{\sigma }_i \circ dW^i_t=0\, , \end{aligned}$$

and the corresponding Itô process is

5.4 $$\begin{aligned} d\varvec{\Pi } + \varvec{\Pi }\times \varvec{\Omega }\, {\text{ d }}t +\frac{1}{2} \sum _i(\varvec{\Pi }\times \varvec{\sigma }_i)\times \varvec{\sigma }_i\, {\text{ d }}t + \sum _i \varvec{\Pi }\times \varvec{\sigma }_i\, dW^i_t= 0\, . \end{aligned}$$

The coadjoint orbit defined by a level set of the quadratic Casimir $\Vert \varvec{\Pi }\Vert ^2=\mathrm {c}^2$ is preserved in our geometrical construction, as may be checked by a direct computation in both the Stratonovich and the Itô stochastic representations. Although the Casimir is conserved, the energy $h(\varvec{\Pi }) = l(\varvec{\Omega })$ is not a conserved quantity in general. Indeed, since the moment of inertia, $\mathbb {I}$ is a symmetric matrix, the stochastic process associated to *h* can be found to be

5.5 $$\begin{aligned} dh= \sum _i(\varvec{\Pi }\times \varvec{\sigma }_i)\cdot [\mathbb {I}^{-1}(\varvec{\Pi }\times \varvec{\sigma }_i)-(\varvec{\Omega }\times \varvec{\sigma }_i) ]\, {\text{ d }}t+2\sum _i (\varvec{\Pi }\times \varvec{\sigma }_i)\cdot \varvec{\Omega }\, dW_t^i\, . \end{aligned}$$

In the general case, one only has bounds for the energy given by the two stable equilibrium positions of the rigid body, namely $E_\mathrm {min} = \frac{1}{2\mathrm {I_3}}|\Pi _3(0)|^2$ and $E_\mathrm {max} = \frac{1}{2\mathrm {I_1}}|\Pi _1(0)|^2$ if $I_1\le I_2\le I_3$. Thus, the energy may randomly fluctuate within these bounds.

Apart from the obvious case of $\mathbb {I}=Id$, one can check that the system with $\mathbb {I}= (I_1,I_1,I_3)$ and $\varvec{\sigma }= (0,0,\sigma _3)$ conserves the energy for every values of $I_1,I_3$ and $\sigma _3$. In this case, the stochastic rigid body reduces to the Kubo oscillator of Kubo et al. (1991)

$$\begin{aligned} d\Pi _1 = \Pi _2(a\Pi _3 {\text{ d }}t + \chi _3\circ dW), \quad d\Pi _2 = -\Pi _1(a\Pi _3 {\text{ d }}t + \chi _3\circ dW)\quad \mathrm {and}\quad d\Pi _3 =0\, , \end{aligned}$$

where $a:= \frac{I-I_3}{I\, I_3}$. This system is integrable by quadratures and a solution is

$$\begin{aligned} \Pi _1(t)&= \Pi _1(0)\, \mathrm {cos}(\gamma t + \chi _3 W_t) - \Pi _2(0)\, \mathrm {sin}(\gamma t + \chi W_t) \\ \Pi _2(t)&= \Pi _2(0)\, \mathrm {cos}(\gamma t + \chi _3 W_t) + \Pi _1(0)\, \mathrm {sin}(\gamma t + \chi W_t)\, , \end{aligned}$$

where $\gamma := a \Pi _3$. Although the deterministic free rigid body is integrable, the only known integrable stochastic rigid body is this particular case.

### The Fokker–Planck Equation

The Fokker–Planck equation of the process (5.3) is simply given for a probability density $\mathbb {P}$ by

5.6 $$\begin{aligned} \frac{\text{ d }}{{\text{ d }}t}\mathbb {P} + (\varvec{\Pi }\times \varvec{\Omega }) \cdot \nabla \mathbb {P} +\frac{1}{2}\sum _i (\varvec{\Pi }\times \varvec{\sigma }_i)\cdot \nabla [(\varvec{\Pi }\times \varvec{\sigma }_i) \cdot \nabla \mathbb {P} ]=0 \,, \end{aligned}$$

where $\nabla$ is the gradient with respect to the independent variable $\varvec{\Pi }\in \mathbb {R}^3$. According to Theorem 2.5, the stationary distribution $\mathbb {P}_\infty$ is constant on coadjoint orbits, which are spheres.

Based on this result, more can be said about the energy evolution of the stochastic rigid body, without embarking on any deeper studies into the coupled stochastic processes (5.5) and (5.3). For example, by ergodicity of (5.3), the long time average of the stochastic rigid body motion follows the limiting distribution $\mathbb {P}_\infty$. In terms of energy, the distribution is not uniform but will be proportional, at a given energy, to the length of the deterministic trajectory of the rigid body with this energy. The energy will thus randomly oscillate between two bounds, with maximum probability to be near the energy of the unstable equilibrium.

### Double Bracket Dissipation

The double bracket dissipation for the rigid body involves the only Casimir $\Vert \varvec{\Pi }\Vert ^2$ and yields, with noise, the dissipative stochastic process

5.7 $$\begin{aligned} d\varvec{\Pi } + \varvec{\Pi }\times \varvec{\Omega }\, {\text{ d }}t + \theta \, \varvec{\Pi }\times ( \varvec{\Pi }\times \varvec{\Omega })\, {\text{ d }}t+ \sum _i\varvec{\Pi }\times \varvec{\sigma }_i \circ dW^i_t=0 \, . \end{aligned}$$

The Itô formulation is similar to (5.4) and will not be written here. The corresponding the Fokker–Planck equation is

5.8 $$\begin{aligned} \begin{aligned} \frac{\text{ d }}{{\text{ d }}t}\mathbb {P}&+ (\varvec{\Pi }\times \varvec{\Omega })\cdot \left( \nabla \mathbb {P} - \theta \, \varvec{\Pi } \times \nabla \mathbb {P}\right) +\frac{1}{2}\sum _i (\varvec{\Pi }\times \varvec{\sigma }_i)\cdot \nabla [(\varvec{\Pi }\times \varvec{\sigma }_i) \cdot \nabla \mathbb {P} ]=0\, . \end{aligned} \end{aligned}$$

The Fokker–Planck equation for stochastic rigid body dynamics with selective decay may be found by specialising the general proof given for Theorem 3.4. Indeed, we can rewrite the Fokker–Planck equation as

5.9 $$\begin{aligned} \frac{\text{ d }}{{\text{ d }}t}\mathbb {P} + (\varvec{\Pi }\times \varvec{\Omega }) \cdot \nabla \mathbb {P} + \nabla \cdot \left( \theta \, \varvec{\Pi } \times (\varvec{\Pi }\times \varvec{\Omega }) \mathbb {P} -\frac{1}{2}\sigma ^2\, \varvec{\Pi }\times ( \varvec{\Pi }\times \nabla \mathbb {P} )\right) =0 \,, \end{aligned}$$

where we have used $\nabla \cdot (\varvec{\Pi }\times (\varvec{\Pi }\times \varvec{\Omega }))= 0$. The last term in (5.8) simplifies as

$$\begin{aligned} \sum _i (\varvec{\Pi }\times \varvec{e}_i) [( \varvec{\Pi }\times \varvec{e}_i)\cdot \nabla \mathbb {P}]&= \sum _i (\varvec{\Pi }\times \varvec{e}_i) [( \nabla \mathbb {P} \times \varvec{\Pi })\cdot \varvec{e}_i] = \varvec{\Pi }\times (\nabla \mathbb {P}\times \varvec{\Pi })\, , \end{aligned}$$

since the sum over *i* is simply the decomposition of the vector $( \nabla \mathbb {P} \times \varvec{\Pi })$ into its $\varvec{e}_i$ components. Consequently, the asymptotic equilibrium solution tends to

5.10 $$\begin{aligned} \mathbb {P}_\infty = Z^{-1}e^{-\frac{2\theta }{\sigma ^2}h(\Pi )}\,, \end{aligned}$$

in which the overall sign of the exponential argument is negative, since $\theta >0$.

**Fig. 1:**
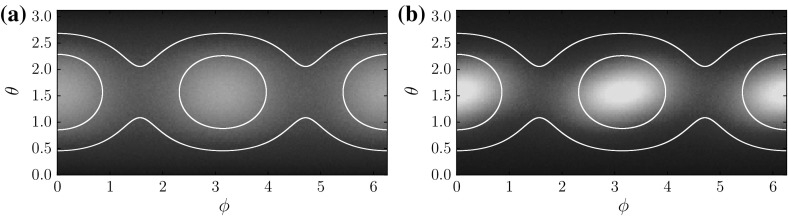
We display two invariant measures of the Fokker–Planck equation of the stochastic free rigid body. We plot in *dark blue* the low density regions and yellow the high density regions. The *left panel* shows a simulation with an isotropic noise which illustrates the Gibbs measure and the *right panel* shows a non-isotropic noise with a near Gibbs measure. The *white lines* show a few deterministic trajectories of the rigid body. **a** Isotropic noise with ${\varvec{\sigma }} \propto (1,0,0),\varvec{\sigma } \propto (0,1,0)$ and $\varvec{\sigma } \propto (0,0,1)$. **b** Non-isotropic noise with $\varvec{\sigma } \propto (1,0,0),$ and $\varvec{\sigma } \propto (0,0,1)$ (Color figure online)

We illustrate the Gibbs measure in Fig. 1a obtained by Monte–Carlo sampling. To illustrate the assumption of isotropic noise necessary to obtain the Gibbs measure, we performed the same simulation but with a non-isotropic noise, displayed on Fig. 1b. The distribution is no longer symmetric, and its analytical form is difficult to obtain. Nevertheless, it remains close to a Gibbs measure.

### Random Attractor

For $\mathfrak {so}(3)$, we can go beyond Theorem 4.5 to obtain the exact value of the sum of the Lyapunov exponents.

#### Proposition 5.2

The sum of the Lyapunov exponents can be given exactly as

5.11 $$\begin{aligned} \sum _i \lambda _i = -3\sigma ^2 - \theta \left( c^2 \mathrm {Tr}\,\mathbb {I}^{-1}-6 \mathbb {E}_\infty h\right) \, , \end{aligned}$$

where *c* is the value of the Casimir function, and $\theta >0$.

#### Proof

We can compute the term *A* of Theorem 4.5 exactly with the structure constants $c^{ij}_k= \epsilon _{ijk}$

$$\begin{aligned} -A(\varvec{\Pi },\varvec{\Pi })&=-\mathrm {Tr}(\mathrm {ad}_\Pi \mathrm {ad}_\Pi \mathbb {I}^{-1})= -c^{im}_nc^{jn}_m\mathbb {I}^{-1} \Pi _i \Pi _j\\&= \Pi _1^2(\mathbb {I}_2^{-1}+\mathbb {I}_3^{-1}) + \Pi _2^2 ( \mathbb {I}_1^{-1}+\mathbb {I}_3^{-1})+\Pi _3^2 (\mathbb {I}_1^{-1}+\mathbb {I}_2^{-1})\,, \end{aligned}$$

which, when combined with the Hamiltonian, yields the result in equation (5.11). $\square$

**Fig. 2:**
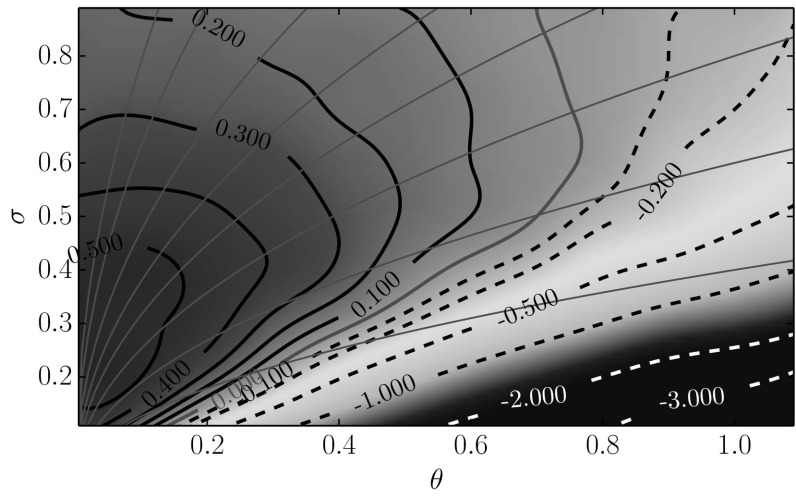
This figure displays the value of the top Lyapunov exponent of the stochastic damped rigid body with $\mathbb {I}= (1,2,3)$ and $c=1$ in the parameter space $(\theta ,\sigma )$. This clearly shows a large region of positive Lyapunov exponents, implying chaotic behaviour in the system. See the text for a more detailed discussion

We now turn to the numerical estimation of the top Lyapunov exponent for the stochastic damped rigid body. More explanation of the numerical scheme used can be found in the appendix A. The result is displayed in Fig. 2 where we sampled $\theta$ and $\sigma$ with 0.1 steps and used a spline interpolation for smoothing data. This result must not be taken to be exact, since, for example, the regions of large or small noise are the least accurate, as larger noise requires smaller time steps and a sufficiently small noise loses the ergodicity property sooner, as the simulations are run for a finite time. Nevertheless, these results numerically demonstrate that the top Lyapunov exponent is positive over a large region of the parameter space $(\theta ,\sigma ^2)$. Based on these numerical results, it is of course not possible to show that the observed chaos is not transient, but the longer runs suggest that the positive top Lyapunov exponent reaches a stable constant value.

The green iso-temperature lines (higher temperatures on the left) in Fig. 2 are tangent to the iso-top-lyapunov lines in the upper left quadrant, where the top Lyapunov exponent is positive. Perpendicular isolines indicate that these two variables can be used as a coordinate system in this figure. Thus we can change from $(\theta ,\sigma )$ to $(\lambda _+,T)$. In fact, we already know that we can use $(\lambda _+,\lambda _-)$ as a coordinate system, which means that in the upper left region of the plot we have proportionality, $\lambda _-\propto T$.

Apart from demonstrating that the top Lyapunov exponent can be positive, this figure also provides us with a better understanding of this system, which we now discuss briefly.

- For $\theta = 0$, the attractor is the entire space, that is the momentum sphere, and the stationary distribution is uniform on it. No random attractor or SRB measure exists in this case. Nevertheless, we learn that by increasing the noise, we first observe an increase of the amount of chaos, and then a decrease. The decrease for large noise is rather slow, and it is not expected that the top Lyapunov exponent will ever become negative under further increase of the noise. This is because the shear of the rigid body is bounded by the difference between the two opposite moment of inertia. In turn, the magnitude of the top Lyapunov exponent is bounded to the extent that it is linked to this shear. This is in contrast with random attractors in the plane, or more generally in non-compact spaces, which can possess an arbitrarily large shear. For example, in the case of planar systems with limit cycles studied numerically by Lin and Young (2008) and analytically by Engel et al. (2016), the top Lyapunov is not bounded for large noise amplitudes.
- The limit $\sigma \rightarrow 0$ cannot be numerically computed, but it is easy to extrapolate it from this graphic. First, notice that if $\theta =\sigma =0$, we are in the Hamiltonian case of rigid body dynamics; hence, the sum of the Lyapunov exponents must be zero and the top Lyapunov exponent is therefore positive. Furthermore, the Lyapunov exponents will depend on the initial conditions, as the system is not ergodic anymore, so the limit $\sigma \rightarrow 0$ is not very well defined. Nevertheless, if $\theta >0$, the top Lyapunov exponent converges to a single negative value.
- Upon examining the plots for various values of the Lyapunov exponents, one notices that the dark region of negative Lyapunov exponent varies rapidly with the parameters $\theta$ and $\sigma$, whereas the rest of the plot shows slower variations. It is interesting to remark that the slope of the line $\lambda ^+=-1$, for example, is close to 0.4, which is smaller than 1. That means that to balance an increase in the noise for some value, the damping must be increased by a larger value.

To conclude, in light of this numerical result, and along with the theorem for the existence of SRB measures, provided $\varvec{\sigma }_i$ spans $\mathbb {R}^3$, the proper dissipation of energy will imply the existence of a non-singular random attractor.

**Fig. 3:**
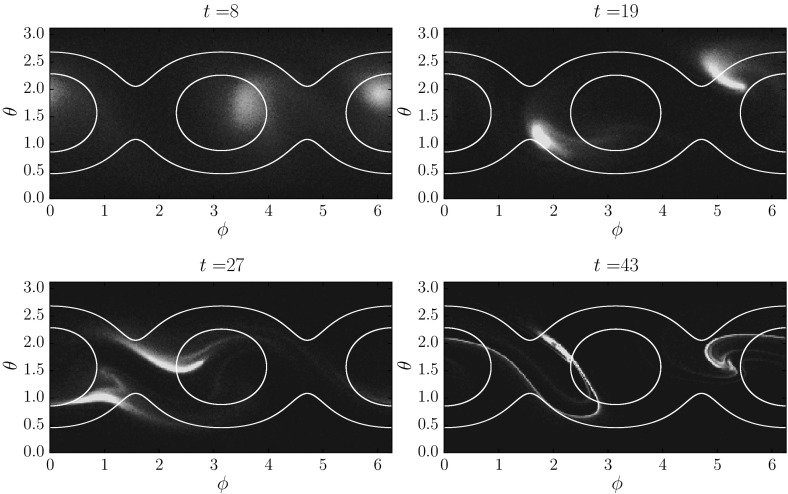
The four panels display snapshots of the same rigid body random attractor with $\mathbb {I}= \mathrm {diag}(1,2,3)$, $\theta =0.5$ and $\sigma = 0.5$. The simulation started with a uniform distribution of rigid bodies on the momentum sphere and created finer and finer structures. The *colour* is in log scale, and we simulated 400,000 rigid body initial conditions with a split-step scheme, see “Appendix A”

The final analysis at this stage of the investigation concerns the nature of the random attractors of this system. From numerical simulations, we display in Fig. 3 a realisation of a random attractor of the rigid body.^1^

The plots in Fig. 3 show the SRB measure, in log scale and exhibit the phenomena of stretching and folding, typical of strange attractors with positive and negative Lyapunov exponents. The positive exponent produces the stretching mechanism, and the negative one produces the folding process. Asymptotically in time, these mechanisms may create a fractal structure, similar to the Smale horseshoe structure for the Duffing attractor. The mechanisms for the creation of the rigid body random attractor can be understood from the underlying rigid body dynamics. The heteroclinic orbits, linking the two saddle equilibrium points which correspond to the direction of the second moment of inertia are the longest orbits, with the fastest dynamics along them. The speed of the motion for each orbit then decreases to reach the stable equilibrium points, associated with the largest or smallest moment of inertia. This change in speed creates a shear on a given non-singular set evolving with the rigid body dynamics. Together with the compactness of the sphere, the combination of noise and dissipation produces the complicated structure of the attractor. One can remark that the attractor of the Duffing oscillator is similar and provides a good example in the deterministic context of the creation of these structures.

A more detailed study of this random attractor will certainly be interesting but is out of the present scope of this work as it would require deeper dynamical systems analysis. Nevertheless, we will briefly study in the next section a simplification of this model which considers periodic kicking instead of Brownian motion.

### Periodic Kicking

We finish this section devoted to the stochastic three-dimensional rigid body by making the simple replacement of the noise by a periodic kicking. It turns out that this type of forced dynamical systems also shows chaotic behaviours, and furthermore the theoretical understanding of this phenomenon is more advanced that for the pure noise case. We refer to Wang and Young (2003) and Lin and Young (2008) and references therein for such studies.

The periodic kicking is achieved by simply replacing the noise $dW^i_t$ by the sum of Dirac delta functions

5.12 $$\begin{aligned} dW_t^i\Rightarrow \sigma _i \sum _{n=1}^\infty \delta ( t- nT)\, , \end{aligned}$$

where *T* is the period for the kicking and $\sigma \in \mathbb {R}^3$ represents the amplitude and direction of the kick, as in the noisy system. It is interesting to remark that the kicking corresponds to a rotation around the fixed axis $\sigma$ and with an angle proportional to $\Vert \sigma \Vert$. Notice that in the noisy case, the axis of rotation was also random, so it was not relevant to consider it for our studies. The double bracket dissipation term is still there; thus, some attractors are expected to emerge, but they will surely not be random. In fact, due to the periodic kicking, the notion of attractor must be modified slightly. Recall that in the noisy case, to have a fixed attractor in time, we needed the notion of pullback attractor. Here, we will fix the attractor by just observing it at discrete times *nT*. Indeed, between each kick, the dynamics relaxes following the damped deterministic rigid body equation.

We will not attempt a theoretical study of this system but rather illustrate its complexity with the help of numerical investigations. Since the parameter space is rather large, we will just highlight the most typical behaviour of the system while only varying the amplitude of the kick, $\Vert \sigma \Vert$ for fixed $T=1$, $\theta = 0.2$, $\sigma = \Vert \sigma \Vert (1,1,1)$ and $\mathbb {I} = \mathrm {diag}(1,2,3)$. The fact that $\sigma$ is not aligned with any eigenvalue of $\mathbb {I}$ makes the configuration of the rigid body generic enough for the present study. Remarkably, this simple system can undergo many different types of dynamical behaviour by simply varying the kicking amplitude $||\sigma ||$.

**Fig. 4:**
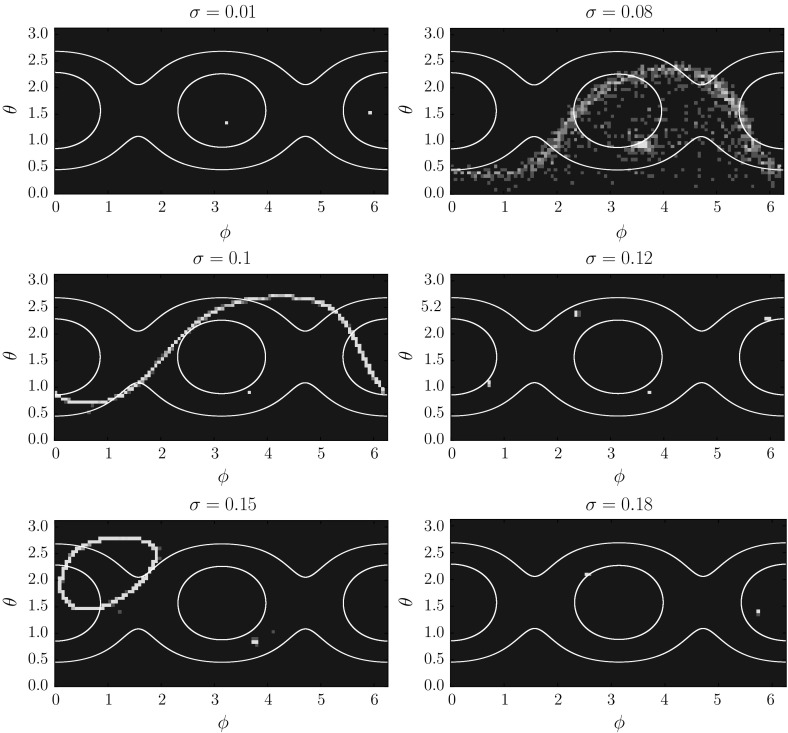
This figure displays several attractors of the periodically kicked rigid body for the different regimes parametrised by the kicking amplitude $\Vert \sigma \Vert$ as described in the text. Each snapshot is taken after the system has reached an equilibrium position, except for $\Vert \sigma \Vert =0.8$. In this case, we have illustrated the transient limit cycle structure which quickly disappears as the solution converges to a singular attractor. For $\sigma =0.12$, the three singular attractors (except the *lower right one*) which are slightly thicker are in fact a single attractor, and the kicking makes these points periodically switch amongst each other. The *last panel* with $\sigma =0.18$ shows a similar behaviour for a single attractor composed of two points that switch between each other

We have numerically scanned the attractors for various values of $\Vert \sigma \Vert$ and have displayed the results in Fig. 4, which we will analyse qualitatively below.

The first thing to remark to understand these different types of behaviour is that the kick will rotate the momentum of the rigid body in the same direction as the original rigid body flow for the lower right region, and in the opposite direction for the upper left region in Fig. 4. For this reason, the effect of the periodic kicking will be different on the opposite sides of the sphere, and this will lead to two different types of attractors near these regions. We now describe the different types of attractors which appear in this system, upon varying the kicking amplitude. Let $\mathcal A^+$ denote the attractor where the dynamical flow and the kicking are in the same direction and let $\mathcal A^-$ denote the other attractor. They will be understood as being the same if only one attractor emerges. Recall that all the parameters are fixed, except the kicking amplitude $\Vert \sigma \Vert$. We thus classify the different regimes according to an ordered sequence $\epsilon _0<\ldots <\epsilon _5$ of values of $\Vert \sigma \Vert$, that we will find later.

**Fig. 5:**
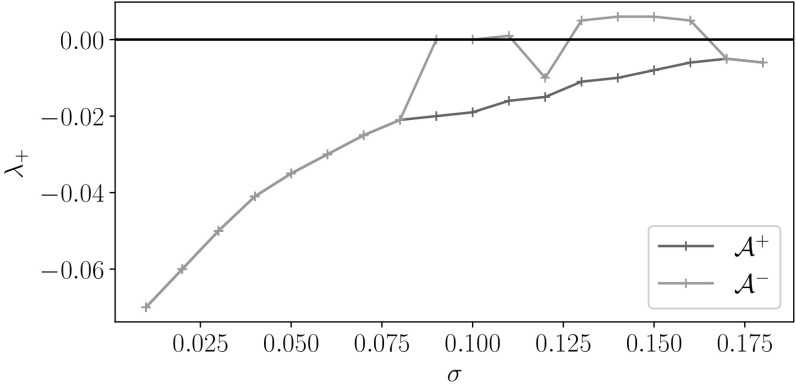
We display the result of the numerical computation of the top Lyapunov exponent for the kicked rigid body as a function of the kicking amplitude $\Vert \sigma \Vert$ for the possibly two attractors. The $\mathcal A^+$ attractor is always singular and thus always has a negative top Lyapunov exponent, whereas the other attractor sometimes shows chaos on the limit cycle

The analysis was done by looking at individual trajectories of rigid bodies and the values of the Lyapunov exponents, which we display in Fig. 5. From all this data, we can estimate the values of the $\epsilon$ in the ordered sequence as approximatively (0.02, 0.08, 0.11.13, 0.16), as illustrated in Fig. 5.

The different regimes are as follow.

1. $\Vert \sigma \Vert < 0.02$: Both attractors $\mathcal A^+$ and $\mathcal A^-$ are singular and near the original rigid body equilibrium positions.
2. $0.02<\Vert \sigma \Vert <0.08$: The points near the region of the previous $\mathcal A^-$ eventually reach the $\mathcal A^+$ region to form one singular attractor near the previous $\mathcal A^+$. Before the collapse of the point near $\mathcal A^-$ to the region near $\mathcal A^+$, we observe a short transient chaos, close to being a limit cycle. The chaos is revealed by a positive Lyapunov exponent during this period.
3. $0.08<\Vert \sigma \Vert <0.11$: The attractor $\mathcal A^-$ is a limit cycle which passes near both original equilibrium of minimum energy and thus is driven by the kicks on both sides of the sphere. The chaos on this limit cycle is not clear from the Lyapunov exponent computation, as the top Lyapunov exponent is very close to 0, see Fig. 5. The other attractor $\mathcal A^+$ is singular, near its previous position.
4. $0.11<\Vert \sigma \Vert <0.13$: The attractor $\mathcal A^-$ consists of 3 singular points at roughly equal distance from the kicking axis $(-1,-1,-1)$. These three points form a single attractor as the kick makes then periodically switch between themselves. The other attractor $\mathcal A^+$ is still singular.
5. $0.13<\Vert \sigma \Vert <0.17$ : The attractor $\mathcal A^-$ consists of a chaotic limit cycle centred around the kicking axis $(-1,-1,-1)$ and $\mathcal A^+$ is still singular. As compared to the previous limit cycle, this one remains near the region where the kicking is opposite to the flow direction and has a stronger chaos, as seen from the Lyapunov exponent computation, see Fig. 5.
6. $0.17<\Vert \sigma \Vert <0.18$: The last region explored here shows that both attractors merge to a single attractor that consists of two fix points. The periodic kicking switches one to the other, as in case (4).

From these findings, the most remarkable result is not the existence of chaos on the limit cycles, but rather the existence of the limit cycles themselves. The chaos can be understood in the same way as for the stochastic case, namely by the shear of the system. The existence of a stable limit cycle is in fact rather subtle as it requires a fine balance between the kicking, the shear and the damping of the rigid body. A precise analytical estimation for the emergence of such limit cycles is of course out of the scope of this work, and we leave it for further studies.

## Semidirect Product Example: The Stochastic Heavy Top

The classic example of semidirect product motion is the heavy top, which arises in the presence of gravity when the support point of a freely rotating rigid body is no longer at its centre of mass. The starting phase space for the heavy top is $T^*\mathrm{SO}(3)$, just as for the free rigid body. When the support point is shifted away from the centre of mass, gravity breaks the symmetry, and the system is no longer $\mathrm{SO}(3)$ invariant. Consequently, the motion can no longer be written entirely in terms of the body angular momentum $\varvec{\Pi }\in \mathfrak {so}(3)^*$. One also needs to keep track of the unit vector $\varvec{\Gamma }$, the “direction of gravity” as seen from the body $(\varvec{\Gamma } = \mathbf {R}^{-1}\mathbf {k}$ where the unit vector $\mathbf {k}$ points upward in space and $\mathbf {R}$ is the element of $\mathrm{SO}(3)$ describing the current configuration of the body). The variable $\varvec{\Gamma }$ may be identified with elements in the coset space *SO*(3) / *SO*(2), where *SO*(3) is the symmetry broken by introducing a special vertical direction for gravity, and *SO*(2) is the remaining symmetry. This *SO*(2) is the isotropy subgroup of *SO*(3) corresponding to rotations around the unit vector $\mathbf {k}$ which leave the direction of gravity invariant.

### The Stochastic Heavy Top

The Lagrangian for the heavy top is the difference between the kinetic energy and the work against gravity, where the fixed vector $\varvec{\chi }$ represents the position of the centre of mass of the body with respect to the fixed point. In body coordinates, the reduced Lagrangian is

6.1 $$\begin{aligned} l(\varvec{\Omega }, \varvec{\Gamma }) = \frac{1}{2} \varvec{\Omega }\cdot \mathbb {I}\varvec{\Omega } - mgl\varvec{\Gamma }\cdot \varvec{\chi }\, . \end{aligned}$$

We refer to see Holm et al. (1998) and Marsden and Ratiu (1999) for a complete description of the semidirect product reduction for the heavy top, which we will not explain here. The stochastic potential will be taken to be linear in both the $\varvec{\Gamma }$ and $\varvec{\Pi }$:

6.2 $$\begin{aligned} \Phi _i(\varvec{\Gamma },\varvec{\Pi }) = \varvec{\sigma }_i\cdot \varvec{\Pi }+\varvec{\eta }_i\cdot \varvec{\Gamma }\,, \end{aligned}$$

where $\varvec{\sigma }_i$ and $\varvec{\eta }_i$ need not span $\mathbb {R}^3$. The stochastic process describing the stochastic heavy top is then

6.3 $$\begin{aligned} \begin{aligned}&d\varvec{\Pi } +(\varvec{\Omega } {\text{ d }}t + \sum _i \varvec{\sigma }_i\circ dW^i_t) \times \varvec{\Pi } +mgl (\varvec{\Gamma } \times \varvec{\chi }){\text{ d }}t+\sum _i mgl( \varvec{\Gamma } \times \varvec{\eta }_i)\circ dW^i_t =0 \,,\\&\quad d\varvec{\Gamma } +(\varvec{\Omega } {\text{ d }}t + \sum _i\varvec{\sigma }_i\circ dW^i_t)\times \varvec{\Gamma }= 0 \,, \end{aligned} \end{aligned}$$

and the corresponding Itô process is

6.4 $$\begin{aligned} \begin{aligned}&d\varvec{\Pi } + (\varvec{\Omega } {\text{ d }}t + \sum _i \varvec{\sigma }_i dW^i_t) \times \varvec{\Pi } + (\varvec{\Gamma }\times mgl\varvec{\chi }){\text{ d }}t \\&\quad +\, \sum _i mgl( \varvec{\Gamma }\times \varvec{\eta }_i)\circ dW^i_t-\frac{1}{2}\sum _i\varvec{\sigma }_i\times (\varvec{\sigma }_i\times \varvec{\Pi }){\text{ d }}t=0 \,,\\&\quad d\varvec{\Gamma } +(\varvec{\Omega } {\text{ d }}t + \sum _i\varvec{\sigma }_i dW^i_t)\times \varvec{\Gamma } -\frac{1}{2} \sum _i\varvec{\sigma }_i\times (\varvec{\sigma }_i\times \varvec{\Gamma }){\text{ d }}t=0 \,. \end{aligned} \end{aligned}$$

The two Casimirs of the heavy top are conserved, $\Vert \varvec{\Gamma }\Vert ^2=k$ and $\varvec{\Pi }\cdot \varvec{\Gamma }=c$. However, the energy is not conserved, as it satisfies the following stochastic process

6.5 $$\begin{aligned} \begin{aligned} \frac{\text{ d }}{{\text{ d }}t} E&= \frac{1}{4} \sum _i \left[ \varvec{\Omega }\cdot (\varvec{\sigma }_i\times (\varvec{\sigma }_i\times \varvec{\Pi })) + \varvec{\Pi }\cdot \mathbb {I}^{-1} ( \varvec{\sigma }_i\times (\varvec{\sigma }_i\times \varvec{\Pi }))\right] {\text{ d }}t\\&\quad +\, \frac{1}{2} \sum _i \left[ (\varvec{\Pi }\times \varvec{\sigma }_i)\cdot \mathbb {I}^{-1} (\varvec{\Pi }\times \varvec{\sigma }_i) - mgl (\varvec{\sigma }\times \varvec{\Gamma })\cdot (\varvec{\chi }\times \varvec{\sigma }_i) \right] {\text{ d }}t \\&\quad +\, \frac{1}{2} \sum _i\left[ \varvec{\Omega }\cdot (\varvec{\Pi }\times \varvec{\sigma }_i) + \varvec{\Pi }\cdot \mathbb {I}^{-1} (\varvec{\Pi }\times \varvec{\sigma }_i) + 2 \varvec{\chi }\cdot (\varvec{\Gamma }\times \varvec{\sigma }_i)\right] dW^i_t \,. \end{aligned} \end{aligned}$$

The energy being only bounded from below, this stochastic process can lead to arbitrary large value for the energy, over a long enough time.

### The Integrable Stochastic Lagrange Top

When $\mathbb {I}$ is of the form $\mathbb {I}=\mathrm {diag}(I_1,I_1,I_3)$ and $\chi =(0,0,\chi _3)$, the deterministic heavy top is called the Lagrange top and is integrable. The integrability comes from the extra conserved quantity $\varvec{\Pi }\cdot \varvec{\chi }$, in this case. For noise, the stochastic process for this quantity is

6.6 $$\begin{aligned} \frac{\text{ d }}{{\text{ d }}t}(\varvec{\Pi }\cdot \varvec{\chi }) = -\frac{1}{2} \sum _i(\varvec{\chi }\times \varvec{\sigma }_i)\cdot (\varvec{\sigma }_i\times \varvec{\Pi })\, {\text{ d }}t - \sum _i\varvec{\chi }\cdot (\varvec{\Pi }\times \varvec{\sigma }_i)dW_t^i\, , \end{aligned}$$

which is not a conserved quantity in general. However, the form of this equation implies that if one selects $\varvec{\sigma }_i=\varvec{\chi }$ then $\varvec{\Pi }\cdot \varvec{\chi }$ is a conserved quantity. It is remarkable that with this choice of noise, the energy is also a conserved quantity, as one can check from equation (6.5). We thus have a stochastic integrable Lagrange top, with a stochastic Lax pair given by

6.7 $$\begin{aligned} d(\lambda ^2\varvec{\chi } + \lambda \varvec{\Pi } + \varvec{\Gamma }) = ((\lambda \varvec{\chi } + \varvec{\Omega } ){\text{ d }}t + \varvec{\chi }\circ dW)\times (\lambda ^2\varvec{\chi } + \lambda \varvec{\Pi } + \varvec{\Gamma })\ , \end{aligned}$$

where $\lambda$ is arbitrary and called a spectral parameter. We refer to Ratiu (1981) for more details about the integrability of the Lagrange top. Following the framework of integrable hierarchies, further developed for infinite dimensional integrable hierarchies in Arnaudon (2015), there exists another integrable stochastic Lagrange top where the stochastic potential is the same as the Hamiltonian. The explanation for the integrability is straightforward, as the change of variable $t\rightarrow t+W_t$ maps the stochastic Lagrange top to the deterministic one; so we will not discuss it in more detail here.

We want to study this stochastic system further, as integrability means that an explicit solution can be found. Indeed, from the standard theory of the heavy top, see for example Arnol’d (1989) and Audin (1996), the equation for $\Gamma _3$ can be found to be of the form $\dot{\Gamma }_3^2= f(\Gamma _3)$, where *f* depends only on the constants of motion *k* and *c*. Then, a straightforward calculation with Euler angles gives

**Fig. 6:**
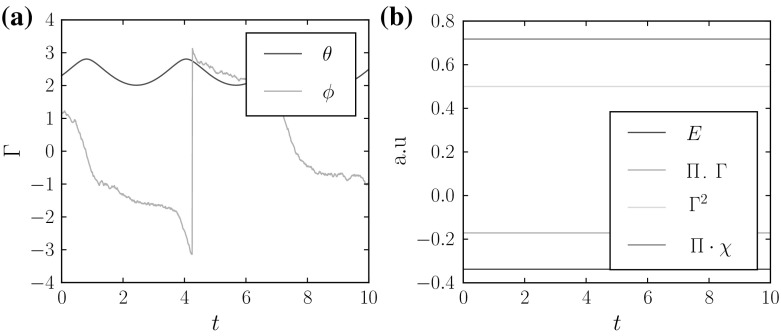
This figure displays a realisation of the motion of the integrable stochastic Lagrange top. The conserved quantities are displayed in the *right panel*. **a** $\Gamma$ motion, **b** conserved quantities

6.8 $$\begin{aligned} \begin{aligned} \dot{\psi }&= \frac{c-k\Gamma _3 }{(1- \Gamma _3^2) I}\\ d \phi&= \left[ \frac{c}{I_3\Gamma _3} - \frac{c-k\Gamma _3 }{I_3\Gamma _3(1- \Gamma _3^2) I} ((1-\Gamma _3^2) I - I_3\Gamma _3^2)\right] {\text{ d }}t -\chi _3\circ dW\,, \end{aligned} \end{aligned}$$

where $\mathrm {cos}(\theta )= \Gamma _3$ gives the third Euler angle. Surprisingly, only $\phi$ has a stochastic motion, while $\psi$ and $\theta$ follow the deterministic Lagrange top motion. This is illustrated in Fig. 6 via a numerical integration of the stochastic Lagrange top equations. The conservation of all the Lagrange top quantities is reproduced, as well as the fact that the noise only influences the $\phi$ component of the Euler angles.

### The Fokker–Planck Equation and Stationary Distributions

We now analyse the associated Fokker–Planck equation for the stochastic heavy top, which is given by

6.9 $$\begin{aligned} \begin{aligned} \frac{\text{ d }}{{\text{ d }}t}\mathbb {P}(\varvec{\Pi }, \varvec{\Gamma })&= (\varvec{\Pi }\times \varvec{\Omega })\cdot \nabla _\Pi \mathbb {P} + (\varvec{\Gamma }\times \varvec{\Omega })\cdot \nabla _\Gamma \mathbb {P} \\&\quad +\, \sum _i\frac{1}{2}(\varvec{\Pi }\times \varvec{\sigma }_i)\cdot \nabla _\Pi \left[ (\varvec{\Pi }\times \varvec{\sigma }_i)\cdot \nabla _\Pi \mathbb {P} \right] \\&\quad +\, \sum _i \frac{1}{2} (\varvec{\Gamma }\times \varvec{\sigma }_i)\cdot \nabla _\Gamma \left[ (\varvec{\Gamma }\times \varvec{\sigma }_i)\cdot \nabla _\Gamma \mathbb {P}\right] \\&\quad +\, \sum _i\frac{1}{2}(\varvec{\Pi }\times \varvec{\sigma }_i)\cdot \nabla _\Pi \left[ (\varvec{\Gamma }\times \varvec{\sigma }_i)\cdot \nabla _\Gamma \mathbb {P} \right] \\&\quad +\, \sum _i\frac{1}{2} (\varvec{\Gamma }\times \varvec{\sigma }_i)\cdot \nabla _\Gamma \left[ (\varvec{\Pi }\times \varvec{\sigma }_i)\cdot \nabla _\Pi \mathbb {P}\right] \,, \end{aligned} \end{aligned}$$

where in our notation $\nabla _\Pi$ denotes the gradient with respect to the $\varvec{\Pi }$ variable only and similarly for $\nabla _{\Gamma }$. By using the semidirect product Lie–Poisson structure of the heavy top

6.10 $$\begin{aligned} \{H,G\}_{HT} := \begin{bmatrix} \nabla _\Pi H&\nabla _\Gamma H \end{bmatrix} \begin{bmatrix} \varvec{\Pi }\times&\varvec{\Gamma } \times \\ \varvec{\Gamma }\times&0 \end{bmatrix} \begin{bmatrix} \nabla _\Pi G\\ \nabla _\Gamma G \end{bmatrix}\, , \end{aligned}$$

the Fokker–Planck equation (6.9) can be written in the double bracket form

6.11 $$\begin{aligned} \frac{\text{ d }}{{\text{ d }}t}\mathbb {P} = \{ h,\mathbb {P}\}_{HT} +\frac{1}{2} \{\Phi ,\{\Phi ,\mathbb {P} \}_{HT}\}_{HT}\, , \end{aligned}$$

where $h(\varvec{\Pi }, \varvec{\Gamma })$ is the Legendre transform of (6.1).

Recall that the stationary marginal distribution on the $\Gamma$ sphere is constant. We study here the distribution in the $\Pi$ coordinate, following the general argument of Theorem 2.6, which gives the bound

6.12 $$\begin{aligned} 0\le \Vert \Pi \Vert (t)\le \Vert \Pi _0\Vert + (mglc) t\, . \end{aligned}$$

**Fig. 7:**
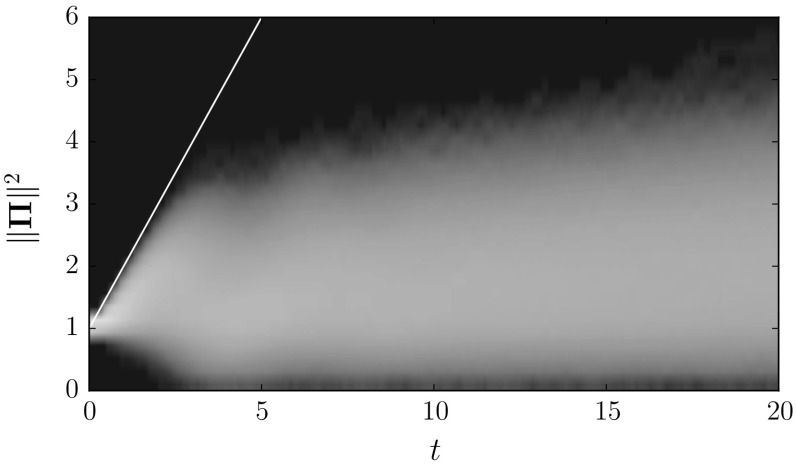
We display the probability distribution of the norm of the momentum of the heavy top, as a function of time. The distribution tends to 0 as time goes to $\infty$, but only linearly as shown by Eq. (6.12) and the white line in this Figure. The expansion is larger for small times, as the distribution is not yet uniform on the angles of the momentum but linearly bounded in time. After this rapid early expansion, the diffusion slows considerably

This bound increases linearly with time and is unbounded only when $t\rightarrow \infty$. This effect is clearly illustrated in the Fig. 7 where the probability distribution of $\Vert \Pi \Vert ^2$ is plotted. The initial conditions are uniform distributions on the $\Gamma$ sphere and a single position for all the momentum, with unit norm. Our system parameters are $m=g=c=1$. Consequently, the linear bound is directly proportional to the time. According to Fig. 7, the bound is reached almost immediately in the first stage of the diffusion, where the $\Gamma$ and $\Pi$ sphere are not yet uniformly covered. After this first short temporal regime, however, the diffusion rate slows considerably below this linear bound.

### Random Attractor

The dissipative heavy top equations can be computed directly from the semidirect theory (see also Bloch et al. 1996) and in Stratonovich form they read, when the Casimir $\varvec{\Pi }\cdot \varvec{\Gamma }$ is used,

6.13 $$\begin{aligned} \begin{aligned}&d\varvec{\Pi } +(\varvec{\Omega } {\text{ d }}t + \sum _i \varvec{\sigma }_i\circ dW^i_t) \times \varvec{\Pi } +mgl (\varvec{\Gamma } \times \varvec{\chi }){\text{ d }}t \\&\quad +\,\theta \varvec{\Gamma }\times (\varvec{\Omega }\times \varvec{\Gamma }){\text{ d }}t +\theta \left[ mgl\varvec{\Pi }\times (\varvec{\chi }\times \varvec{\Gamma }) - \varvec{\Pi }\times (\varvec{\Pi }\times \varvec{\Omega })\right] {\text{ d }}t= 0 \,,\\&\quad d\varvec{\Gamma } + (\varvec{\Omega } {\text{ d }}t + \sum _i\varvec{\sigma }_i\circ dW^i_t)\times \varvec{\Gamma } + \theta \left[ mgl\varvec{\Gamma }\times (\varvec{\chi }\times \varvec{\Gamma }) - \varvec{\Gamma }\times (\varvec{\Pi }\times \varvec{\Omega })\right] {\text{ d }}t = 0. \end{aligned} \end{aligned}$$

Notice that the two Casimirs which define the coadjoint orbits are preserved by both the noise and the dissipation, as expected. Also, recall the form of the deterministic energy decay

6.14 $$\begin{aligned} \frac{dh}{{\text{ d }}t}&= -\theta \left\| \varvec{\Omega } \times \varvec{\Gamma } \right\| ^2 -\,\theta \left\| \varvec{\Omega }\times \varvec{\Pi } + mgl \varvec{\chi } \times \varvec{\Gamma } \right\| ^2 \,, \end{aligned}$$

which was used earlier to prove the existence of the random attractor after a nonlinear change of variables. The other Casimir $\Vert \varvec{\Gamma }\Vert ^2$ can also be used to derive dissipative equations, but energy dissipation will be slower, as only the first term in (6.14) and the first decay term of the $d\varvec{\Pi }$ are left. The equilibrium solution of the purely dissipative system are found by setting the right-hand side of (6.14) to 0 and are always of the form $\varvec{\Omega }= \varvec{\Gamma }= \varvec{\chi }$ if $\varvec{\chi }$ is an eigenvalue of $\mathbb {I}$. If not, the equilibrium is aligned to another direction that we will not compute here, as we will stick to the simple generic case.

We can compute the lower bound for the value of the sum of the Lyapunov exponents using Theorem 4.7 to find

**Fig. 8:**
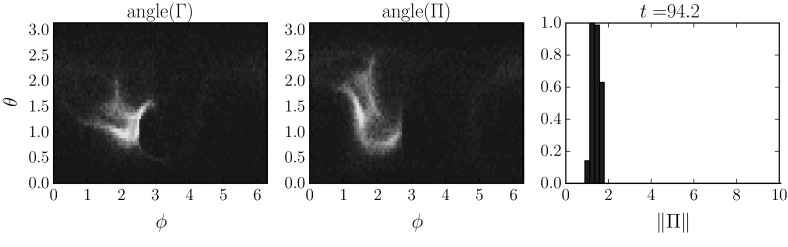
We display here three projections of the attractor on the coadjoint orbit of the heavy top at two different times. The *left panel* is a projection on the sphere $\Vert \Gamma \Vert ^2=1$, the *second panel* on the sphere $\Vert \Pi \Vert ^2= 1$ and the *third panel* is the amplitude of the momentum. We used $\theta = 0.2$, $\sigma =0.1$, $\mathbb {I}= \mathrm {diag}(1,2,3)$, $g=1$ and 20, 000 realisations of the stochastic heavy top, with initial conditions uniformly distributed on a subset of the coadjoint orbit defined by $\Vert \Pi \Vert ^2= 1$

6.15 $$\begin{aligned} \sum _i \lambda _i \ge -6\sigma ^2 -\theta c^2 \mathrm {Tr}(\mathbb {I}^{-1})\,, \end{aligned}$$

which is always negative. We will not study here the parameter space of the top Lyapunov exponent as done for the rigid body, but only display two instances of an attractor of the heavy top in Figure 8.^2^ The formation of the attractors seems not to be of horseshoe type, as occurs for the rigid body. This may be explained by the higher dimensionality of the coadjoint orbit (dimension 4) on which the attractor is supported.

## Two Other Examples

This section briefly sketches two other stochastic symmetry-reduced examples of the present theory which follow immediately from the examples of the *SO*(3) rigid body and the heavy top, treated in the previous sections. These are the *SO*(4) rigid body and the spring pendulum.

### The *SO*(4) Rigid Body

For a complete study of the rigid body motion on *SO*(4) we refer to Birtea et al. (2012) and references therein. We use the generic elements

$$\begin{aligned} X = \begin{pmatrix} 0 &{} x_1 &{} x_2 &{} x_3 \\ -x_1 &{} 0 &{} x_4 &{} -x_5 \\ -x_2 &{} -x_4 &{} 0 &{} x_6\\ -x_3 &{} x_5 &{} -x_6 &{} 0 \end{pmatrix}\, , \end{aligned}$$

or $X= (X_1,X_2)\in \mathbb {R}^6$. In terms of vectors $(X_1,X_2)\in \mathbb {R}^6$ and $(X_1',X_2')\in \mathbb {R}^6$ we have

$$\begin{aligned}{}[(X_1,X_2),(X_1',X_2')] = \left( X_1\times X_1' + X_2\times X_2', X_1\times X_2' + X_2\times X_1'\right) \, . \end{aligned}$$

The coadjoint action is the same, under the trace-pairing. The Casimir for *SO*(4) are given by

$$\begin{aligned} C_1&= \mathrm {Tr}(X^2)=\sum _i x_i^2= \Vert X_1\Vert ^2+\Vert X_2\Vert ^2 \,,\\ C_2&= \sqrt{\mathrm {det}(X)}= x_1x_6 + x_2x_5 + x_3x_4= X_1\cdot X_2 \,. \end{aligned}$$

The first Casimir is a 4-dimensional sphere and the second is the Pfaffian, or scalar product between two vectors. The momentum-velocity relation is $\Pi = J\Omega + \Omega J$ where $J= \mathrm {diag}(\lambda _1,\ldots ,\lambda _6)$ and the Hamiltonian $H(\Pi )= \frac{1}{2} (\Pi _1\cdot \Omega _1 + \Pi _2\cdot \Omega _2)$. We thus have the following stochastic 4-dimensional rigid body equations

7.1 $$\begin{aligned} \begin{aligned} d (\Pi _1,\Pi _2)&= \left( \Pi _1\times \Omega _1 + \Pi _2\times \Omega _2, \Pi _1\times \Omega _2 + \Pi _2\times \Omega _1\right) {\text{ d }}t \\&\quad +\, \sum _i \left( \Pi _1\times \sigma _1^i + \Pi _2\times \sigma _2^i, \Pi _1\times \sigma _2^i + \Pi _2\times \sigma _1^i\right) \circ dW_i\,, \end{aligned} \end{aligned}$$

which preserve the coadjoint orbit.

We now look at the selective decay term for the Casimir $C_2(\Pi )= \Pi _1\cdot \Pi _2$. This term reads, upon using semi-simplicity,

$$\begin{aligned} SD&= \mathrm {ad}_{(\Pi _2,\Pi _1)}\mathrm {ad}_{(\Pi _2,\Pi _1)} (\Omega _1, \Omega _2)\\&=\left( \Pi _2\times (\Pi _2\times \Omega _1 + \Pi _1\times \Omega _2) + \Pi _1\times (\Pi _2\times \Omega _2 + \Pi _1\times \Omega _1),\right. \\&=\left( \Pi _2\times \Pi _2\times \Omega _1 +\Pi _2\times \Pi _1\times \Omega _2 + \Pi _1\times \Pi _2\times \Omega _2 + \Pi _1\times \Pi _1\times \Omega _1,\right. \\&\quad \left. \Pi _2\times \Pi _2\times \Omega _2 + \Pi _2\times \Pi _1\times \Omega _1 + \Pi _1\times \Pi _2\times \Omega _1 + \Pi _1\times \Pi _1\times \Omega _2 \right) . \end{aligned}$$

One can directly check that the first Casimir $C_1$ is also preserved by this flow.

#### Proposition 7.1

This stochastic dissipative *SO*(4) free rigid body admits a random attractor.

#### Proof

This is a direct application of the theory developed in Sect. 3. $\square$

The stationary distribution will be centred around the minimal energy position, associated with the direction of the maximal moment of inertia. We will not numerically investigate the random attractors for this system here. However, further theoretical studies are indeed possible, and these would be interesting to discuss elsewhere, especially the integrable case, with a particular choice of the noise.

### Spring Pendulum

From the heavy top equation, one can derive the spherical pendulum by letting one of the components of the diagonal inertia tensor in body coordinates tend to zero, e.g., $I_3\rightarrow 0$. This follows, because the spherical pendulum is infinitely thin and, hence, does not have any inertia for rotations around its axis. We shall choose $\mathbb {I}= \mathrm {diag}(I,I,\epsilon )$ in the heavy top equations and then take the limit $\epsilon \rightarrow 0$ so that the dynamics on $\Pi _3$ vanishes. The similarity of this system with the rigid body allows us to consider an extension of the spherical pendulum which is called the spring pendulum (Lynch 2002). To include the dynamics of the length of the spring pendulum, we introduce a new variable $R(t)\in \mathbb {R}\setminus \{0\}$ and enforce its dynamical evolution in the variational principle by adding $P(\dot{R}- v ){\text{ d }}t$ where *v* denotes the velocity of the mass along the pendulum, and *P* denotes its associated momentum. The Lagrangian is then found to be

7.2 $$\begin{aligned} l(\varvec{\Omega },\varvec{\Gamma }, R,v) = \frac{m}{2}R^2\varvec{\Omega }\cdot \mathbb {I}\varvec{\Omega } - mgR\varvec{\Gamma }\cdot \varvec{\chi }+ \frac{1}{2} m v^2 - \frac{k}{2}(R - 1)^2 \,, \end{aligned}$$

where $\varvec{\chi }$ represents the initial position of the pendulum which is taken to be (0, 0, 1) in accordance with our choice of inertia tensor. In (7.2), we denote the spring constant by *k* and the mass of the pendulum by *m*.

We shall assume a general linear stochastic potential of the form,

7.3 $$\begin{aligned} \Phi (\varvec{\Pi },\varvec{\Gamma },R,P):= \varvec{\sigma }\cdot \varvec{\Pi } + \varvec{\eta }\cdot \varvec{\Gamma }+ \alpha R + \beta P \,, \end{aligned}$$

for constant vectors $\varvec{\sigma },\varvec{\eta },$ and constant scalars $\alpha ,\beta$. Consequently, the stochastic spring pendulum equations are given by

7.4 $$\begin{aligned} \begin{aligned} d\varvec{\Pi }&= \varvec{\Pi }\times \varvec{\Omega } {\text{ d }}t + mgR \varvec{\Gamma } \times \varvec{\chi } {\text{ d }}t + \varvec{\Pi }\times \varvec{\sigma }_i\circ dW_t^i + \varvec{\Gamma }\times \varvec{\eta }_i\circ dW_t^i \\ d\varvec{\Gamma }&= \varvec{\Gamma }\times \varvec{\Omega } {\text{ d }}t + \varvec{\Gamma } \times \varvec{\sigma }_i\circ dW_t^i \\ dR&= \frac{P}{m |\varvec{\chi } |^2}{\text{ d }}t + \beta dW_t \\ dP&= -m g \varvec{\Gamma }\cdot \varvec{\chi } {\text{ d }}t - k(R-1) |\varvec{\chi } |^2 {\text{ d }}t +\frac{1}{mR^3} \varvec{\Pi }\cdot \mathbb {I}^{-1} \varvec{\Pi }- \alpha dW_t \,. \end{aligned} \end{aligned}$$

The analysis above is valid, provided $\epsilon >0$ in the inertia tensor. In the limit $\epsilon \rightarrow 0$, we may set $\varvec{\Omega }_3=0$ and thereby recover the stochastic elastic spherical pendulum equations.

The equation set in (7.4) consists of two parts: the stochastic heavy top equations, coupled to a pair of stochastic canonical Hamilton equations for the (*R*, *P*) variables. The coupling between the two subsets of equations occurs through the dependence on *R* together with $\varvec{\Omega }$ and $\varvec{\Gamma }$ in the Lagrangian (7.2).

The Fokker–Planck equation is now easily derived, and it reads

7.5 $$\begin{aligned} \begin{aligned} \frac{\text{ d }}{{\text{ d }}t}\mathbb {P}&= \{H,\mathbb {P}\}_{HT}+ \{H,\mathbb {P}\}_{\mathrm {can}} + \frac{1}{2} \{\Phi ,\{\Phi ,\mathbb {P}\}_{HT} \}_{HT} \\&\quad +\, \{\Phi ,\{\Phi ,\mathbb {P}\}_{HT} \}_{\mathrm can} + \frac{1}{2} \{\Phi ,\{\Phi ,\mathbb {P}\}_{\mathrm can } \}_{\mathrm can} \,, \end{aligned} \end{aligned}$$

where $\{\,\cdot \,,\, \cdot \,\}_{\mathrm {can}}$ is the canonical Poisson bracket with respect to the (*R*, *P*) variables. The coupling between the elastic and pendulum motions is too complicated to extract any information from the Fokker–Planck equation. Indeed, inspection of the motion on (*R*, *P*) shows that the advection equation for (*R*, *P*) depends on the other variables. This inextricable complex dependence precludes finding the limiting distribution explicitly, despite the simple Laplacian form of the diffusion operator.

As pointed out by Lynch (2002), the deterministic elastic spherical pendulum system is a toy model for the lowest modes of atmosphere dynamics. For this application, the motion of the spring oscillations encoded in *R* is considerably faster than the pendulum motion and smaller in amplitude. Averaging the deterministic Lagrangian over the relatively rapid oscillations of the spring yields a nonlinear resonance between the modes of a type which also appears in the atmosphere. The noise can be included in either of the two types of dynamics, and each will influence the other through the nonlinear coupling. Also, for small oscillations around the equilibrium, the deterministic nonlinear coupling produces star-shaped orbits (Holm and Lynch 2002; Lynch 2002), which can be perturbed, or even entirely destroyed, by the introduction of the noise, depending on its amplitude.

## Conclusion and Open Problems

Before stating some open problems arising from this work, we will briefly summarise it. In the first section, we reviewed and developed the new machinery of stochastic geometric mechanics, in the context of finite dimensional systems which admit a group of symmetry of semi-simple type. The main results emerged from the introduction of a particular type of noise that preserves the coadjoint motion of the deterministic equations. The associated Fokker–Planck equation was found to possess interesting geometrical properties, related to the Lie–Poisson formulation of the equation of motion. The Lie–Poisson formulation was used to derive its stationary solution which is constant on the level set of the Casimirs prescribed by the initial conditions. The second section was devoted to the introduction of dissipation with the double bracket term, for which the coadjoint orbits are still preserved by the flow of the equation. This particular combination of multiplicative noise and nonlinear dissipation on coadjoint orbits yields non-constant invariant measures often referred to as Gibbs measures. This type of invariant measure makes an interesting connection to statistical physics and naturally, provides us with a notion of temperature for these systems. The second outcome of the noise-dissipation interaction is the existence of so-called random attractors, which are mathematical objects deeply connected to the theory of random dynamical systems. We demonstrated, by adapting the standard tools from the random dynamical system theory, that such objects do exist in the mechanical systems studied here. Furthermore, we gave conditions on the dissipation and noise amplitudes for the existence of non-singular such attractors which will in turn support an SRB measure. The next two sections were devoted to the application of this theory to the standard examples in geometric mechanics, which are the free rigid body and the heavy top for the semidirect product extension, also developed here. We studied these explicit stochastic processes in detail, and in particular with illustrative numerical simulations. The final section touched upon other related examples such as a spring pendulum which can be viewed as an extension of the heavy top with a direct product structure, and a higher dimensional rigid body, written on *SO*(4).

We now end by listing several open problems which have been formulated during the course of this work.

- Some of the results presented here relied on the assumption of compactness of the coadjoint orbits; for example, in estimating the sum of the Lyapunov exponents, and for the study of the stationary distributions. This assumption is probably unnecessary, but properly addressing its removal would require more advanced mathematical tools than we have used here.
- We were only able to obtain a numerical demonstration that the top Lyapunov exponent is positive. This numerical demonstration could be made considerably more refined, and possibly analytical results could also be derived in future studies.
- We only touched upon the analysis of random attractors via numerical simulations, but much more may be said about these objects by, for example, studying their Lyapunov exponents in more detail, and studying the underlying dynamical process of their formation. This is motivated by the fact that even in the two simple illustrative examples of random attractors treated here, we observed two rather different solution behaviours.
- Although we restricted ourselves to semisimple Lie algebras, we were able to write most of the equations for more general Lie algebras. Hence, the present line of reasoning should be valid for other similar systems by modifying the proofs accordingly. Examples of such systems would include semidirect products with arbitrary advected quantities, solvable or nilpotent Lie algebras, the Toda lattice and possibly infinite dimensional Lie groups such as the diffeomorphism group.

## Appendix A: Numerical Scheme

Here we briefly discuss our numerical scheme for the integration of the stochastic rigid body equations. The heavy top being a direct extension of this scheme, we will not treat it here.

We use a split-step scheme where the deterministic part of the equation is integrated with the Python solver *odeint* of Scipy that used the classic scheme *lsoda*. The stochastic term is integrated via the exact solution of $d\varvec{\Pi } = \varvec{\Pi } \times \circ \mathbf {dW}$ where $\mathbf {dW}= \sum _i \varvec{\sigma }_i dW_t^i$, as

A.1 $$\begin{aligned} \varvec{\Pi }(t+{\text{ d }}t) = e^{\widehat{ \mathbf {dW}}} \varvec{\Pi }(t)\, , \end{aligned}$$

where $\widehat{\mathbf {dW}}$ is corresponding anti-symmetric matrix, or $\mathfrak {so}(3)$ Lie algebra element, and the exponential is the matrix exponential.

The computation of the top Lyapunov exponent is done with this scheme extended to also compute the evolution of the linearisation, $\delta \Pi$. The form of the equation for $\delta \Pi$ is similar to the rigid body equation, and the previous split-step algorithm is implemented in the same way. Despite having a good preservation of the coadjoint orbit for the original stochastic process, this scheme does not accurately preserve the restriction of the linearisation to be tangent to the coadjoint orbit. The scheme *lsoda* being implicit, we need an interpolation between $\Pi (t)$ and $\Pi (t+{\text{ d }}t)$, which is achieved by a spherical linear interpolation, or *slerp*. Even with this interpolation, the condition $\delta \Pi \cdot \Pi =0$ is not precise enough and leads to an incorrect estimate of the norm $\Vert \delta \Pi \Vert$ in the computation of the Lyapunov exponent. We thus project out the part of $\delta \Pi$ aligned with $\Pi$ at each time step such that $\delta \Pi$ remains in the tangent space to the coadjoint orbit. This projection gives coherent results, but it affects the convergence of the different estimations of the top Lyapunov exponent. Namely, for each different initial condition, the corresponding top Lyapunov exponent will take more time to converge to its true value. We overcome this effect by averaging over several realisations of the top exponent computation, which is 50 in our case.

## Footnotes

1 See http://wwwf.imperial.ac.uk/~aa10213/ for a video of this random attractor.

2 See http://wwwf.imperial.ac.uk/~aa10213/ for a video of this random attractor.
